# A Silk-Derived Dual Peptide System Suppresses Skin Photoaging by Inhibiting PDGFRβ-Mediated Cellular Senescence and TRPV4-Mediated Melanogenesis

**DOI:** 10.34133/research.1255

**Published:** 2026-05-08

**Authors:** Yueting Sun, Quan Zhang, Yajun Hou, Rui Huang, Ligang Jiang, Qiang Chen, Zhaohua Fang, Zhaoming Dong, Ping Zhao, Qingyou Xia

**Affiliations:** ^1^ Integrative Science Center of Germplasm Creation in Western China (Chongqing) Science City, Chongqing Technology Innovation Center of Breeding, Biological Science Research Center, Southwest University, Chongqing 400715, China.; ^2^ Shanghai Pechoin Cosmetics Co. Ltd., Shanghai 201400, China.

## Abstract

The utility of silk protein spans from East Asian traditions to promising applications in modern biomaterials science. While its bioactive effect is widely accepted, its molecular mechanisms and the supporting data have remained elusive. This study reveals the anti-photoaging mechanism of silk protein enzymatic hydrolysate (SEH), identifying key peptides SO1 from silk seroin and SC1 from silk sericin. Mechanistically, SO1 targets PDGFRβ to inhibit the NF-κB/ERK/SASP cellular senescence axis, while SC1 targets TRPV4 to suppress MITF-driven melanin synthesis. Additionally, the SO1 and SC1 combination suppresses key hallmarks of photoaging. It reduced senescence, confirmed by diminished β-galactosidase activity and SASP, while concurrently mitigating oxidative stress and hyperpigmentation, and promoting cell migration. Further network pharmacology analysis reveals a multi-component interaction network connecting bioactive silk peptides, target receptors, and downstream signaling pathways involved in mitigating photoaging. This integrative mechanism was defined as the “Silk Peptides Mesh”, with SO1 and SC1 identified as representative core peptides within this system. Finally, a 28-d skin clinical trial involving 30 participants confirms these findings, showing that a cream containing SO1 and SC1 improves photoaging-related skin indicators. Importantly, this work establishes a paradigm shift from conventional single-molecule approaches to a multi-component network understanding of bioactive peptide functionality.

## Introduction

Aging is increasingly understood as a dual process, driven by both intrinsic biological clocks and cumulative environmental damage, and interconnected through the widely accepted hallmarks of aging [1,2]. It is clear that the progression of aging can be pharmacologically modulated [3], and important research efforts have been dedicated to discover the bioactive compounds that can mitigate age-related decline [4]. Among the various extrinsic stressors, ultraviolet (UV) radiation is a primary factor that accelerates aging, particularly in the skin [5]. Prolonged UV exposure induces oxidative stress, inflammation, and aging responses, resulting in pathologies collectively known as photoaging. These detrimental effects can demonstrate as skin disorders, such as actinic keratosis, and increase the risk of skin cancers [6]. Current treatments for photoaging, such as tretinoin and vitamin C, often cause side effects like skin irritation and photosensitivity [7]. Therefore, a critical need exists to identify novel bioactive compounds for photoaging that may also hold therapeutic potential for other age-related diseases.

Against the backdrop of the rising global trend in utilizing natural protein resources [8,9], natural bioactive peptides are garnering increasing attention as promising anti-photoaging agents due to their multifunctionality and biocompatibility. Silk protein has gained broad recognition as a versatile biomaterial, with extensive applications and increasing research interest in tissue engineering and diverse clinical fields [10–14]. Silk protein consists of approximately 75% fibroin and 25% sericin, supplemented by enzymes, protease inhibitors, and bioactive small molecules [15]. Beyond its conventional use in the textile industry, silk has a long history in East Asia for the topical treatment of skin disorders, which is now supported by modern reports of its protective, antioxidant, anti-inflammatory, and metabolic regulatory activities. Our recent studies have revealed the anti-inflammatory pathways of hydrolyzed sericin [16], identified the anti-aging peptide TS263, which promotes extracellular matrix (ECM) expression [17], and developed a biosynthetic system to genetically functionalize silk proteins with potential for biomedical applications [18–20].

To further investigate the photoaging-preventive bioactivity and underlying mechanisms of peptides derived from silk protein, this study used a multi-omics approach that combined transcriptomics, proteomics, and network pharmacology. We determined that silk protein enzymatic hydrolysate (SEH) mainly suppressed photoaging through cellular senescence signaling pathway. Consequently, we identified SO1 as the core functional peptide of SEH. SO1 mitigates photoaging by binding to PDGFRβ to inhibit the extracellular signal-regulated kinase (ERK)/nuclear factor κB (NF-κB)/senescence-associated secretory phenotype (SASP) axis. SC1 was identified as the main depigmenting peptide, which binds to TRPV4 to inhibit MITF and subsequently suppress melanin synthesis. Further network pharmacology analysis showed that SEH achieves its anti-photoaging efficacy through what we term the “Silk Peptides Mesh (SPM)”—a multi-component network where bioactive peptides connect with target receptors to regulate multiple signaling pathways and genes involved in mitigating photoaging, with SO1 and SC1 identified as representative core peptides within this system. Finally, a mixture of SO1 and SC1 demonstrated comprehensive efficacy against skin photoaging in clinical tests. Notably, our findings transition the traditional focus on individual peptide molecules toward a systems-level perspective that emphasizes coordinated multi-component interactions in mediating biological effects, offering a guiding framework for the rational design and application of silk peptide-based therapeutics.

## Results

### Preparation of SEH

**Fig. 1.**
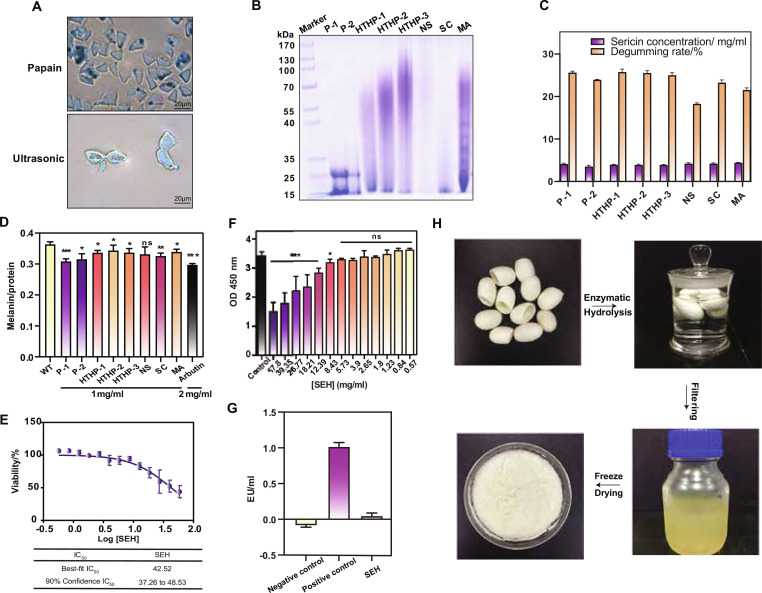
Silk protein extraction. (A) Cross-sectional electron microscope images of silk fibers after degumming by papain and ultrasonic treatment. (B) Molecular weight of sericin extracted from cocoons treated with 12 and 10 h of papain (P-1 and P-2), 2, 1, and 0.5 h of high temperature and high pressure (HTHP-1, HTHP-2, and HTHP-3), neutral soap (NS), sodium carbonate (SC), and malic acid (MA). (C) Comparison of silk protein concentrations (mg/ml) and degumming rates (%) for different samples. (D) Measurement of melanin levels normalized to protein content for various silk protein treatments. Data are presented as mean ± SD (*n* = 6). (E) Cell viability percentage as a function of log concentration of SEH. (F) Optical density (OD) measurements at 450 nm reflecting cell viability at varying concentrations of SEH. Data are presented as mean ± SD (*n* = 6). (G) Endotoxin content detection in different samples. Endotoxin-free water served as the negative control, and 1 EU/ml standard endotoxin served as the positive control. (H) SEH preparation process. Data are presented as mean ± SD (*n* = 3). ns = not significant, *P < 0.05, **P < 0.01, ***P < 0.001.

To create a silk peptide library, we evaluated various extraction methods (Table S1), including papain hydrolysis for 12 and 10 h (P-1 and P-2, respectively), high temperature and high pressure for 2, 1, and 0.5 h (HTHP-1, HTHP-2, and HTHP-3, respectively), and extractions using neutral soap (NS), sodium carbonate (SC), and malic acid (MA). Cryo-electron microscopy confirmed that methods like papain hydrolysis and HTHP were highly efficient at removing the sericin layer from silk fibers, which directly correlated with high extraction rates (also called degumming rates, approximately 25%). In contrast, methods like ultrasonic extraction were ineffective (Fig. 1A and Fig. S1). While several methods showed high extraction rates, sodium dodecyl sulfate–polyacrylamide gel electrophoresis (SDS-PAGE) analysis revealed that papain hydrolysis was superior for generating a library of low-molecular-weight peptides, showing the most complete and consistent degradation with components primarily under 25 kDa (Fig. 1B and C). To screen for anti-melanogenic activity, B16F10 melanocytes were treated with each of the 8 sericin libraries. All libraries, except for NS, significantly inhibited melanin synthesis (*P* < 0.05). Notably, the P-1 library, derived from papain hydrolysis, demonstrated the most potent inhibitory effect, surpassing even the positive control, arbutin (Fig. 1D).

Based on its superior peptide profile and bioactivity, the P-1 method was selected to produce SEH for all subsequent experiments. Safety assessments confirmed that SEH has no cytotoxicity in NIH3T3 fibroblasts and no significant impact on cell viability at concentrations below 5.73 mg/ml (Fig. 1E and F). Furthermore, endotoxin testing demonstrated that SEH contained negligible endotoxin levels comparable to the negative control (endotoxin-free water) and significantly lower than the 1 endotoxin unit (EU)/ml positive control, confirming its safety for biological applications (Fig. 1G and Fig. S2). The preparation process involved enzymatic hydrolysis of cocoons followed by filtration and freeze-drying to obtain SEH powder, which was then reconstituted in solution for experimental use (Fig. 1H).

### SEH inhibited melanin synthesis during the photoaging process

**Fig. 2.**
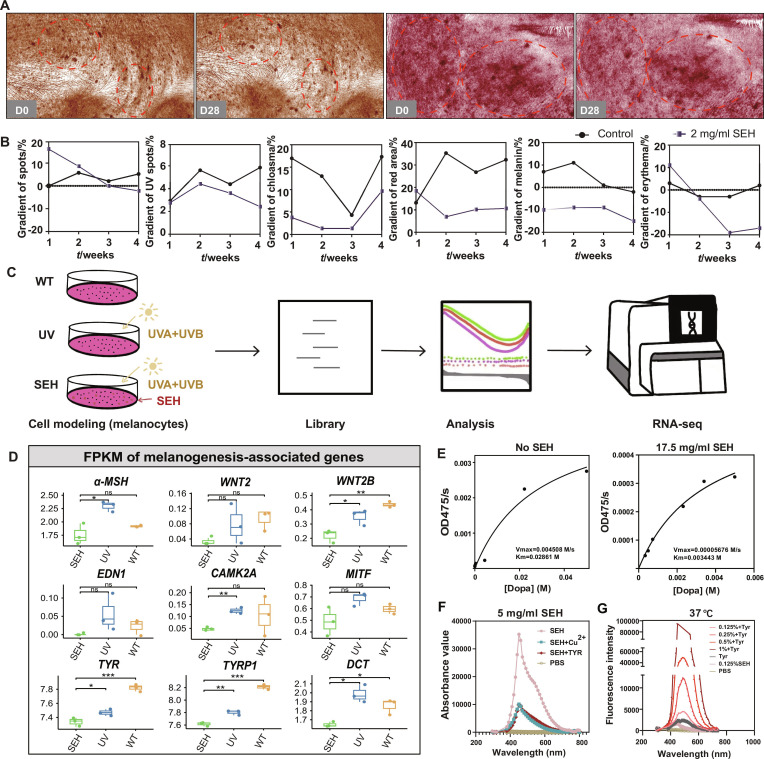
SEH inhibits photoaging and melanogenesis through multi-level mechanisms. (A) Representative skin images at baseline (D0) and after 28 d (D28) of SEH application. (B) Quantitative analysis of various skin parameters over 4 weeks in control and SEH-treated groups. (C) Schematic workflow for the transcriptomic analysis. (D) Fragments per kilobase of transcript per million mapped reads (FPKM) values of key melanogenesis-related genes. ns = not significant, *P < 0.05, **P < 0.01, ***P < 0.001. (E) Michaelis–Menten plot illustrating the enzyme kinetics of TYR in the presence and absence of SEH (17.5 mg/ml). (F) Ultraviolet–visible (UV–Vis) absorption spectra of SEH, tyrosinase (TYR), SEH mixed with TYR, and SEH mixed with copper sulfate (CuSO_2_). (G) Fluorescence intensity profiles for TYR and varying concentrations of SEH at temperatures of 37 °C.

As a preliminary observational pilot study, we conducted a small-scale, double-blind, half-face study to evaluate the skincare efficacy of SEH. A 45-year-old male and a 25-year-old female participant applied a cream containing 2 mg/ml of SEH to one-half of their face for 28 consecutive days, while the contralateral side received a vehicle cream as a control (Table S2). Results demonstrated that SEH significantly improves skin aging. Standardized imaging and time-course analysis over the 4-week period revealed favorable trends in parameters including skin spots, UV spots, chloasma, and red areas, showing that SEH effectively reduced hyperpigmentation and erythema, with treated areas consistently outperforming the control regions (Fig. 2A and B and Fig. S3). These preliminary observations indicated that SEH may be effective in addressing early photoaging phenomena, such as hyperpigmentation and erythema, warranting further investigation in a larger cohort.

To explore the molecular mechanisms of SEH’s anti-photoaging effects, we performed transcriptomic analysis on primary human melanocytes. The cells were divided into 3 groups: an untreated control, a UV-irradiated group (UVA + UVB), and a group pretreated with 1 mg/ml SEH before UV irradiation, followed by RNA-sequencing (RNA-seq) analysis (Fig. 2C). The results showed that SEH significantly down-regulated key melanogenesis-related genes, indicating that SEH inhibits melanin production stimulated by UV radiation (Fig. 2D).

To further understand how SEH works, we tested its direct effect on the key enzyme tyrosinase (TYR). As shown in the Michaelis–Menten plot, we found that SEH is a potent, mixed-type inhibitor of TYR, significantly reducing the enzyme’s maximum reaction speed (*V*_max_) and altering its affinity for its substrate (*K*_m_) (Fig. 2E and Table S3). Spectroscopy analysis confirmed that SEH directly binds to both the TYR enzyme and its essential Cu^2+^ (Fig. 2F), and fluorescence studies showed that this interaction changes the enzyme’s structure (Fig. 2G and Fig. S4). At the cellular level, SEH was as effective as arbutin at inhibiting melanin but proved to be less irritating (Fig. S5). These results showed that SEH suppressed the melanogenesis pathway at both the genetic and protein levels, which contributes to its ability to mitigate UV-induced photoaging.

### SEH inhibited melanocyte photoaging through the cellular senescence signaling pathway

**Fig. 3.**
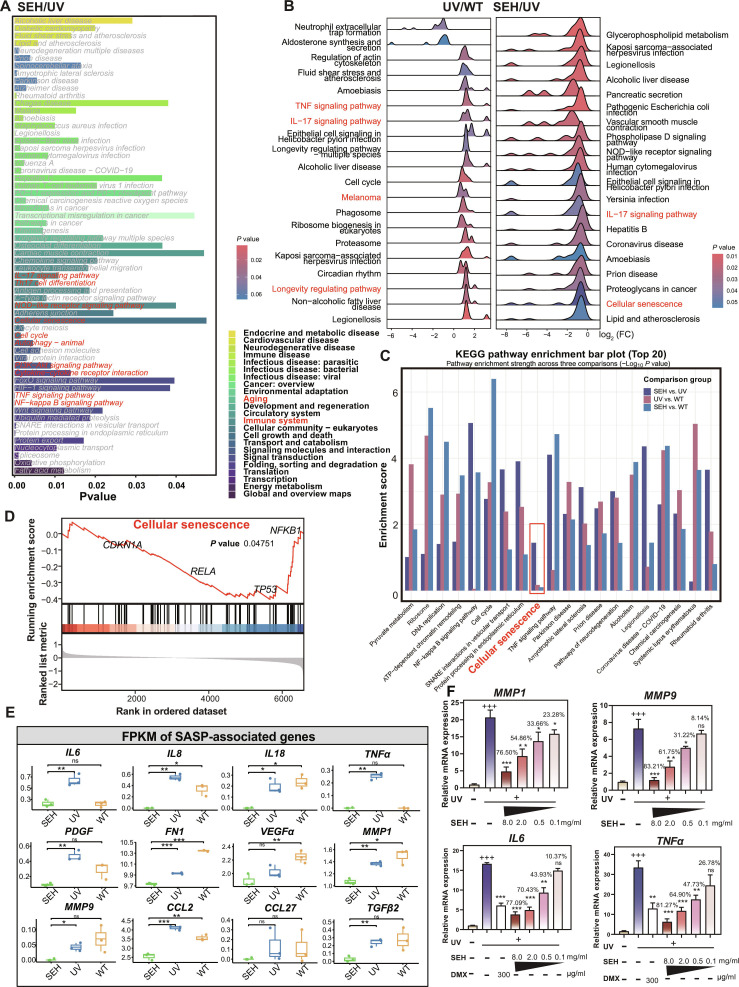
Transcriptomic analysis of SEH’s effect on UV-induced gene expression. (A) Bar plot of KEGG pathway enrichment analysis for differentially expressed genes, comparing the SEH/UV group to the UV/WT group. Bar length corresponds to the *P* value. (B) Ridge plot visualizing differential gene expression patterns between UV/WT and SEH/UV groups in middle expressed gene set. Positive log_2_ (fold change) indicates pathway up-regulation, while negative values indicate down-regulation. (C) Bar plot showing the top 20 enriched KEGG pathways across 3 pairwise comparisons: UV versus SEH, UV versus WT, and SEH versus WT in high expressed gene set. The enrichment score represents the −log_10_ *P* value. (D) GSEA plot for the Cellular Senescence signaling gene set. Key contributing genes are labeled. (E) Boxplots displaying the normalized expression (FPKM) of SASP genes for SEH, UV, and WT groups. (F) qRT-PCR validation of expression changes in MMP1, MMP9, IL6, and TNFα genes. Gene expression levels were normalized to GAPDH. Data are presented as mean ± SD (*n* = 4). “+” compared with the control group, “*” and the inhibition rates compared with the UV group. ns = not significant, *P < 0.05, **P < 0.01, ^+++/^***P < 0.001.

Beyond melanin production, we found that inflammation and senescence were more critical factors in melanocyte photoaging. UV exposure activated numerous inflammation-related signaling pathways, and SEH demonstrated excellent protective effects against these changes (Tables S4 and S5). To understand this better, Gene Set Enrichment Analysis (GSEA)–Kyoto Encyclopedia of Genes and Genomes (KEGG) analysis revealed that the pathways most significantly associated with SEH’s response to UV exposure were related to the immune system and aging (Fig. 3A and Fig. S6). Further analysis confirmed SEH’s protective role by showing that while UV exposure activated pathways involved in inflammation and cellular senescence, SEH treatment strongly suppressed them. This suppressive effect was particularly notable in key pathways such as cellular senescence, tumor necrosis factor (TNF) signaling, and interleukin-17 (IL-17) signaling (Fig. 3B).

Based on the calculation of the enrichment index for different comparison groups, most pathways clustered toward the lower-left region, indicating that SEH treatment activated protective signaling pathways to counteract UV-induced stress. Pathway enrichment scores of middle/high expressed gene sets across different comparison groups revealed substantial alterations in most inflammation-related pathways following SEH treatment, indicating that SEH activated protective signaling cascades to counteract UV-induced stress. Remarkably, the cellular senescence pathway (highlighted in red box) exhibited a consistent pattern of UV-induced activation and SEH-mediated suppression in both middle and high expressed gene sets (Fig. 3B to D). Cellular senescence pathway (highlighted in the red circle) was also the most highly enriched excluding cancer pathways (Fig. S7). This suggested that cellular senescence is a key target for SEH in protecting cells from UV-induced photoaging.

UV radiation causes DNA damage, activates the p53 signaling pathway, and triggers cellular senescence through SASP. In our study, SASP-related genes were also suppressed by SEH, such as the proinflammatory cytokines *IL6*, *IL8*, and *TNFα*, and the matrix metalloproteinase enzymes *MMP1* and *MMP9* (Fig. 3E and F).

Having established that SEH prevents photoaging by targeting both melanogenesis and cellular senescence, we proceeded to investigate the specific bioactive peptides responsible for these dual protective effects and their corresponding receptor-mediated signaling pathways.

### Identification of bioactive peptides in SEH targeting high-expression anti-aging receptors

**Fig. 4.**
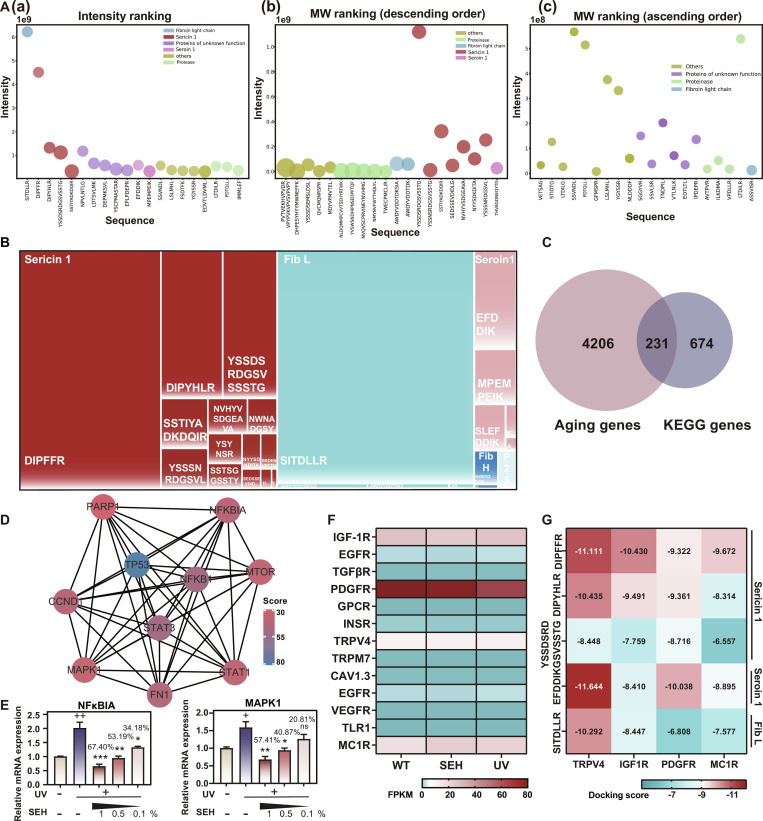
Identification of bioactive peptides in SEH and their interactions with aging-related receptors. (A) Peptidome profiling of SEH extracts showing (a) intensity ranking and molecular weight distribution in (b) descending and (c) ascending order. (B) Treemap visualization of identified peptide sequences in SEH. (C) Venn diagram illustrating the overlap between aging-associated genes from GeneCards/OMIM databases and KEGG pathway genes. (D) PPI network constructed using STRING and Cytoscape. (E) qRT-PCR validation of expression changes in NfκBIA and MAPK1 genes. Gene expression levels were normalized to GAPDH. Data are presented as mean ± SD (*n* = 4). “+” compared with the control group, “*” and the inhibition rates compared with the UV group. ns = not significant, ^+/^*P < 0.05, ^++/^**P < 0.01, ***P < 0.001. (F) Heatmap showing differential expression of senescence-associated membrane receptors across treatment conditions (WT, SEH, UV). (G) Molecular docking analysis heatmap displaying binding energies (kcal/mol) between 5 peptides and the 4 receptors, with stronger interactions indicated by more negative values.

We conducted a peptidomics analysis using liquid chromatography–tandem mass spectrometry (LC-MS/MS), identifying a total of 105 peptides in SEH (Table S6). Peptides were analyzed through intensity ranking and molecular weight ranking (Fig. 4A). A dendrogram was used to visually present the sources and abundances of the peptides (Fig. 4B). The peptides in SEH primarily originated from Sericin 1, Fib L, Seroin 1, Fib H, and P25 proteins. Among the identified peptides, the most abundant were DIPFFR, SITDLLR, DIPYHLR, YSSDSRDGSVSSSTG, and EFDDIK, which were derived from Sericin 1, Fib L, and Seroin 1. These 5 peptides were subsequently subjected to docking studies with potential receptor molecules.

To explain SEH’s anti-aging mechanisms, we overlapped aging-related genes [from GeneCards/Online Mendelian Inheritance in Man (OMIM)] with SEH-regulated KEGG pathway genes, identifying 231 high-confidence targets (Fig. 4C). A protein–protein interaction (PPI) network of these genes revealed a core module with key nodes like including NFKBIA, STAT1, STAT3, TNF, and MAPK8 (Fig. 4D), and the mRNA expression validation confirmed the bioactive effects of SEH (Fig. 4E and Fig. S8). These network-derived genes reinforced that SEH acts by modulating central senescence signaling pathways.

The skin photoaging responses are modulated by key receptors, including the epidermal growth factor receptor (EGFR), platelet-derived growth factor receptor (PDGFR), Toll-like receptors (TLRs), as well as melanocyte-stimulating hormone receptor (MC1R), TNF-α receptor (TNF-αR), and transient receptor potential channel family (TRPV) family channels [21–24]. To systematically identify the most relevant receptor targets for peptide-based intervention, we analyzed the expression profiles of 13 receptors associated with cellular senescence signaling pathways based on our RNA-seq data (Fig. 4F). Among these senescence-related receptors, 4—TRPV4, IGF1R, PDGFRβ, and MC1R—exhibited notably high expression levels in photoaged skin, distinguishing them from the remaining receptors with lower expression. Given that highly expressed receptors are more likely to serve as effective therapeutic targets and mediate biological responses, we selected these 4 receptors as the primary candidates for subsequent molecular docking analysis.

Molecular docking was used to simulate interactions between these 4 receptors and our 5 signature peptides, which were selected based on their highest abundance in MS analysis (Fig. 4G). Peptide–receptor combinations with binding energies below −10 kcal/mol were selected for further molecular docking simulations to explain detailed interaction mechanisms, including IGF1R versus DIPFFR, PDGFRβ versus EFDDIK, TRPV4 versus DIPFFR, TRPV4 versus DIPYHLR, TRPV4 versus EFDDIK, and TRPV4 versus SITDLLR.

### SO1 peptide represented the core anti-aging component in SEH

**Fig. 5.**
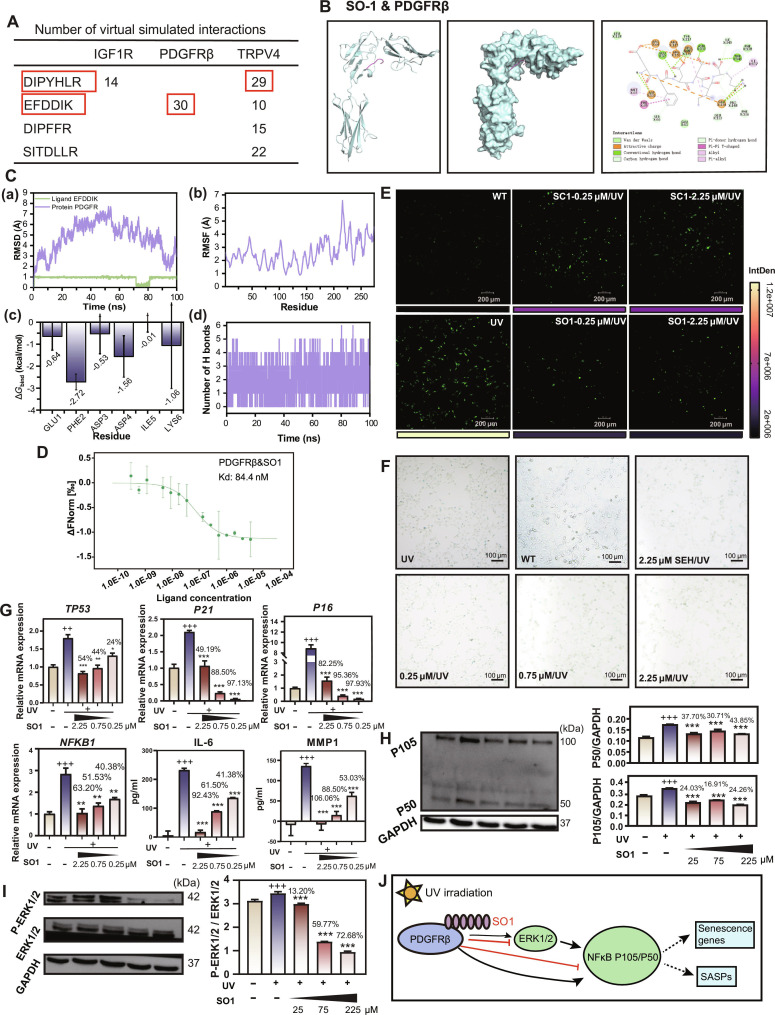
Interaction of SO1 and PDGFRβ and the inhibition mechanism of SO1 on photoaging. (A) Quantification of molecular interactions between selected peptides and receptor targets, with highest interaction pairs DIPYHLR-TRPV4 (29) and EFDDIK-PDGFRβ (30) highlighted in red boxes. (B) Molecular docking analysis of 3-dimensional (3D) and 2D binding modes between SO1 and PDGFRβ. (C) Molecular dynamics simulation of (a) RMSD plot of PDGFRβ with and without ligand during 100-ns simulation, (b) per-residue RMSF analysis, (c) binding free energy calculations for key residues, and (d) hydrogen bond analysis during simulation. (D) MST binding curves showing the interaction between SO1 peptide and PDGFRβ receptor (*K*_d_ = 84.4 nM). Data points represent mean ± SD from independent measurements (*n* = 3), and curves were fitted to determine dissociation constants (*K*_d_). (E) Fluorescence microscopy images showing ROS production (green) in human melanocyte PIG1 with different treatment. Color scale indicates fluorescence intensity. Scale bars, 200 μm. (F) β-Galactosidase activity in human keratinocytes HaCaT under WT, UV, SEH/UV, and SO1/UV treatments. Scale bars, 100 μm. (G) qRT-PCR analysis of TP53, P21, and P16 mRNA expression, and ELISA analysis of IL-6 and MMP1 protein expression in UV-irradiated melanocyte cells treated with different concentrations of SO1. Gene expression levels were normalized to GAPDH. Data are presented as mean ± SD (*n* = 4). “+” compared with the control group, “*” and the inhibition rates compared with the UV group. ns = not significant, ^+/^*P < 0.05, ^++/^**P < 0.01, ^+++/^***P < 0.001. (H and I) WB analysis of inflammatory protein expression in UV-irradiated melanocytes treated with varying concentrations of SO1. GAPDH served as loading control. Data are presented as mean ± SD (*n* = 3). “+” compared with the control group, “*” and the inhibition rates compared with the UV group. ^+++/^***P < 0.001. (J) Schematic diagram of the anti-aging mechanism of SO1.

Based on molecular docking simulations, we quantified the number of interactions between the identified peptides and their potential receptor targets (Fig. 5A and Fig. S9). Among the 5 high-abundance peptides, 2 peptide–receptor pairs demonstrated exceptionally strong interactions. DIPYHLR from Sericin 1 with TRPV4 (29 interactions) and EFDDIK from Seroin 1 with PDGFRβ (30 interactions), including hydrogen bonds, charge attractions, and other interactions (Table S7). These 2 peptides, combining the highest abundance and strongest binding interactions, were selected for further investigation. Given their distinct origins, we designated these peptides as SC1 (Sericin-derived) and SO1 (Seroin-derived).

We conducted detailed characterization of SO1 binding to PDGFRβ. Molecular modeling analysis revealed that the SO1 peptide binds precisely within the PDGFRβ receptor’s pocket, where it is stabilized by a dense network of hydrogen bonds and van der Waals forces between key residues (Fig. 5B). Further, we performed comprehensive molecular dynamics simulations over 100 ns. The root mean square deviation (RMSD) analysis showed minimal fluctuations (around 1Å) for the SO1 peptide (green line), indicating remarkably stable binding, while PDGFRβ (purple line) maintained structural integrity throughout the simulation. Residue-specific flexibility assessment through root mean square fluctuation (RMSF) revealed that most regions remained within 5 Å, demonstrating that SO1 binding stabilized the receptor’s core structure. Binding energy decomposition calculated using molecular mechanics/generalized born surface area (MM/GBSA) yielded a total binding energy of −21.97 ± 1.49 kcal/mol, with key contributions from residues GLU1 (−0.64 ± 0.62), PHE2 (−2.72 ± 0.35), ASP3 (−0.53 ± 0.90), ASP4 (−1.56 ± 0.94), ILE5 (−0.01 ± 0.43), and LYS6 (−1.06 ± 1.96). Throughout the simulation, an average of 2 to 3 hydrogen bonds and a peak of 6 bonds stabilized the complex, confirming their critical role. Time-lapse visualization further showed that SO1 remained stably bound within the PDGFRβ binding cavity for the entire 100-ns simulation (Fig. 5C and Fig. S10).

To validate the direct interaction between SO1 and PDGFRβ, microscale thermophoresis (MST) experiments were conducted. The results demonstrated strong binding affinity between SO1 peptide and PDGFRβ receptor with a *K*_d_ of 84.4 nM (Fig. 5D). The nanomolar-range *K*_d_ values confirm high-affinity interactions, supporting the molecular docking predictions and validating SO1 and PDGFRβ interaction as key molecular mechanisms underlying SO1’s biological activities.

Consistent with the docking results, SO1 demonstrated potent anti-photoaging effects at the cellular level. It effectively suppressed UV-induced generation of reactive oxygen species (ROS) and β-galactosidase, with significant inhibition observed even at a low concentration of 0.25 μM (Fig. 5E and F). Quantificational real-time polymerase chain reaction (qRT-PCR) and enzyme-linked immunosorbent assay (ELISA) analyses first demonstrated that SO1 effectively counteracted the UV-induced up-regulation of core senescence markers TP53, P16, and P21, down-regulated the expression of the NFκB1 gene itself, and markedly reduced the secretion of key SASP factors, including IL6, IL1β, and MMP1 (Fig. 5G and Fig. S11). To further investigate the underlying mechanisms of these effects, we next performed Western blot (WB) analysis. Consistent with the transcriptional and secretory changes, UV irradiation was found to increase the expression of both NF-κB p105 and p50, as well as the phosphorylation of ERK1/2, whereas SO1 treatment markedly attenuated these effects (Fig. 5H and I). Collectively, these findings demonstrate that SO1 attenuates UV-induced photoaging by dismantling a coordinated PDGFRβ/ERK/NF-κB signaling axis, thereby suppressing both the core cellular senescence program and the associated inflammatory SASP (Fig. 5J).

### SC1 peptide represented the core anti-melanogenic component in SEH

**Fig. 6.**
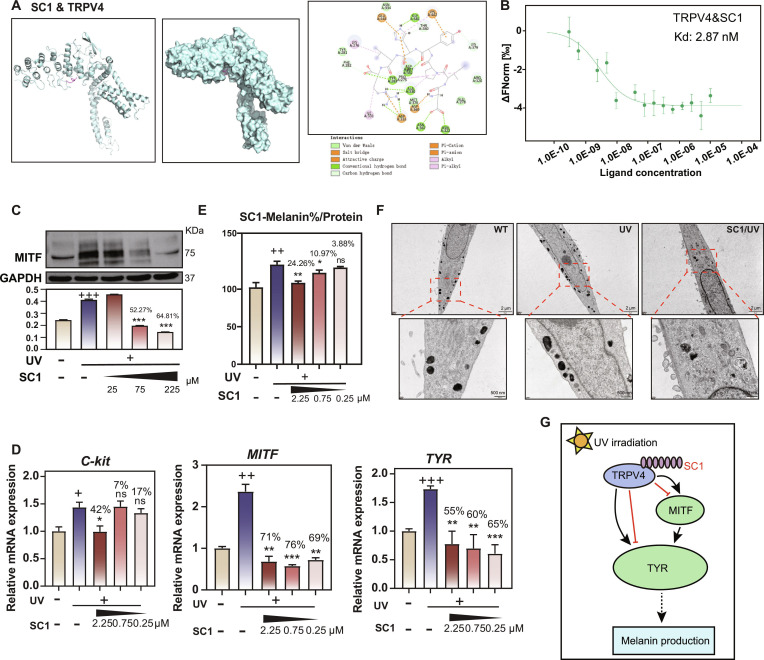
Interaction of SC1 and TRPV4 and the inhibition mechanism of SC1 on melanin production. (A) Molecular docking analysis of 3D and 2D binding modes between SC1 and TRPV4. (B) MST binding curves showing the interaction between SC1 peptide and TRPV4 receptor (*K*_d_ = 2.87 nM). Data points represent mean ± SD from independent measurements (*n* = 3), and curves were fitted to determine dissociation constants (*K*_d_). (C) WB analysis of MITF protein expression in UV-irradiated melanocyte cells treated with different concentrations of SC1. Data are presented as mean ± SD (*n* = 3). “+” compared with the control group, “*” and the inhibition rates compared with the UV group. ^+++/^***P < 0.001. (D) Relative mRNA expression levels of C-kit, MITF, and TYR in melanocytes treated with SC1 and UV. Gene expression levels were normalized to GAPDH. Data are presented as mean ± SD (*n* = 4). “+” compared with the control group, “*” and the inhibition rates compared with the UV group. ns = not significant, ^+/^*P < 0.05, ^++/^**P < 0.01, ^+++/^***P < 0.001. (E) Quantification of melanin content in melanocyte cells treated with varying concentrations of SC1 following UV irradiation. Data are presented as mean ± SD (*n* = 3). “+” compared with the control group, “*” and the inhibition rates compared with the UV group. ns = not significant, *P < 0.05, ^++/^**P < 0.01. (F) Electron microscopy images of melanocytes under WT, UV, and SC1/UV treatments. Scale bars, 2 μm and 500 nm. (G) Schematic diagram of the mechanism of action of SC1.

Meanwhile, we identified SC1 peptide (DIPYHLR) as an anti-melanogenic characteristic component in SEH. First, we performed a virtual structural analysis of interaction between SC1 and TRPV4. The ribbon model and surface representation illustrated the binding conformation, while the interaction map revealed multiple stabilizing forces including van der Waals interactions, salt bridges, conventional hydrogen bonds, and π–cation interactions, collectively explaining the high binding affinity observed (Fig. 6A). To further confirm the specific binding between SC1 and TRPV4, MST assays were performed. The analysis revealed a remarkably high binding affinity, with a *K*_d_ value of 2.87 nM for the SC1 and TRPV4 interaction (Fig. 6B). This nanomolar dissociation constant indicates a robust and specific binding event, corroborating the computational docking results and establishing the SC1–TRPV4 complex as a critical molecular basis for SC1’s functional effects. These diverse interaction types suggested a stable binding complex that could effectively modulate TRPV4 channel activity.

WB analysis and RT-PCR results confirmed the functional consequences of this molecular interaction. WB analysis showed that SC1 reduced MITF protein expression by over 50% compared to UV-treated controls at 75 μM or higher concentration (Fig. 6D). At the transcriptional level, UV exposure significantly up-regulated key melanogenesis regulators including *C-kit*, *MITF*, and *TYR* genes, while SC1 treatment counteracted these changes (Fig. 6C). Most importantly, melanin content analysis showed that SC1 treatment at 2.25 μM restored melanin production to levels comparable with the cell control group, effectively reversing UV-induced melanin overproduction (Fig. 6E). Electron microscopy images also demonstrate the same trend. SC1 treatment maintained normal morphology while significantly reducing melanosome density and maturation (Fig. 6F). These results collectively demonstrated that SC1 effectively inhibited melanogenesis through the TRPV4/MITF signaling cascade (Fig. 6G).

### SO1 and SC1 exert their biological effects through PDGFRβ and TRPV4

**Fig. 7.**
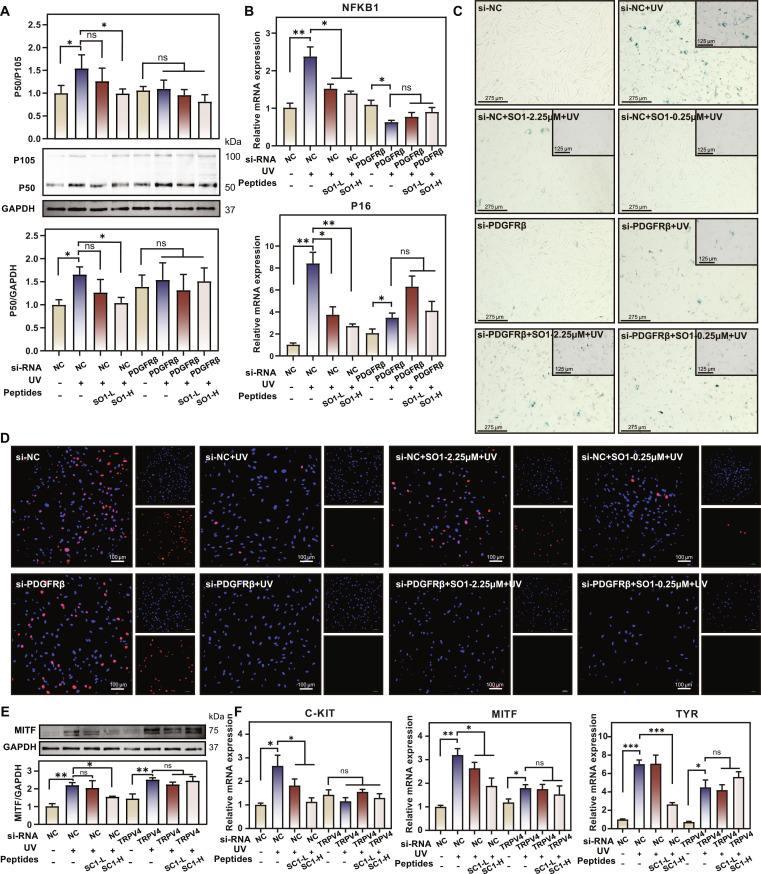
Receptor knockdown experiments. (A) WB analysis of P105/P50 protein expression in fibroblasts treated with si-NC or si-PDGFRβ, with or without UV irradiation and SO1 peptide treatment. Data are presented as mean ± SD (*n* = 3). ns = not significant, *P < 0.05. (B) qRT-PCR analysis of NFKB1 and P16 mRNA expression in fibroblasts under indicated treatment conditions. Gene expression levels were normalized to GAPDH. Data are presented as mean ± SD (*n* = 4). ns = not significant, *P < 0.05, **P < 0.01. (C) Representative images of SA-β-gal staining in fibroblasts transfected with si-NC or si-PDGFRβ, followed by UV irradiation and SO1 peptide treatment. Scale bars, 275 and 125 μm. (D) EdU incorporation assay in fibroblasts under indicated conditions. Scale bars, 100 μm. Insets show separated channels. (E) WB analysis of MITF protein expression in HaCaT cells transfected with si-NC or si-TRPV4, with or without UV stimulation and SC1 peptide treatment. Data are presented as mean ± SD (*n* = 3). ns = not significant, *P < 0.05, **P < 0.01. (F) qRT-PCR analysis of melanogenesis-related genes in HaCaT cells under indicated treatment conditions. Gene expression levels were normalized to GAPDH. Data are presented as mean ± SD (*n* = 4). ns = not significant, *P < 0.05, **P < 0.01, ***P < 0.01.

To establish the direct causal relationship between peptide–receptor binding and the observed downstream signaling effects, we performed receptor knockdown experiments in relevant cell models. For SO1, which targets PDGFRβ, we transfected fibroblasts with small interfering RNA (siRNA) against PDGFRβ (si-PDGFRβ) or negative control siRNA (si-NC), followed by UV irradiation and SO1 peptide treatment. WB analysis demonstrated that while SO1 treatment effectively suppressed UV-induced up-regulation of P105/P50 in si-NC cells, this protective effect was completely abolished in PDGFRβ-depleted cells (Fig. 7A), indicating that PDGFRβ is essential for SO1’s regulatory effect on NF-κB signaling. Consistent with the protein-level findings, PDGFRβ knockdown similarly abolished SO1’s suppressive effects on UV-induced up-regulation of NFKB1 and P16 mRNA expression (Fig. 7B). At the phenotypic level, senescence-associated β-galactosidase (SA-β-gal) staining and 5-ethynyl-2′-deoxyuridine (EdU) incorporation assays further confirmed this receptor dependency: SO1 failed to rescue UV-induced senescence or restore proliferative capacity in PDGFRβ-depleted cells (Fig. 7C and D).

For SC1, which targets TRPV4, we performed analogous experiments in HaCaT keratinocytes, a well-established cell model for studying melanogenesis processes. While SC1 treatment effectively suppressed MITF up-regulation in NC cells, this inhibitory effect was completely lost in TRPV4 knockdown cells (Fig. 7E), demonstrating that TRPV4 is required for SC1’s regulation of MITF. qRT-PCR analysis of melanogenesis-related genes further confirmed this dependency. SC1 treatment significantly down-regulated expression of *C-KIT*, *MITF*, and *TYR* in si-NC cells, but these suppressive effects were abolished upon TRPV4 knockdown (Fig. 7F).

Taken together, these receptor knockdown experiments provide genetic evidence that the biological effects of SO1 and SC1 are mediated through their receptors. The complete abrogation of peptide efficacy upon receptor knockdown confirms that these peptide–receptor interactions represent essential mechanistic links to the observed downstream pathway inhibition and phenotypic outcomes. While the current findings establish the necessity of these receptors, the precise molecular mechanisms warrant further investigation to fully elucidate the peptide–receptor signaling cascade, such as whether SO1’s effects depend on PDGFRβ kinase activity or involve alternative receptor-mediated signaling events.

### Network pharmacology analysis of SEH revealed the integrated signaling network underlying the SPM model

**Fig. 8.**
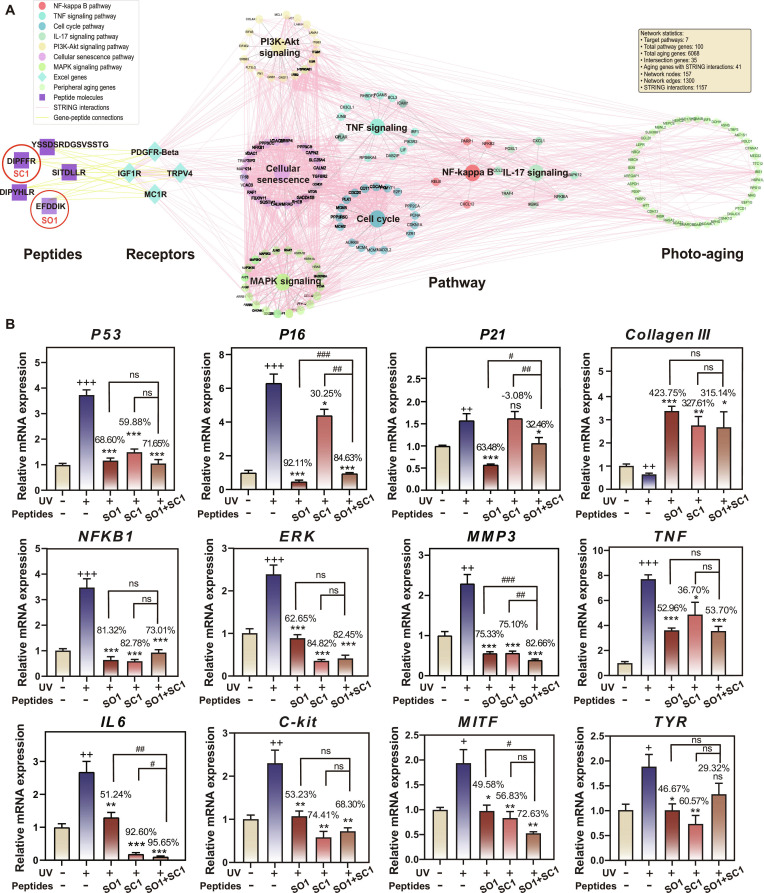
Network pharmacology analysis of SEH. (A) Network pharmacology analysis of SEH revealed the integrated signaling network underlying the SPM model. (B) Relative mRNA expression levels of aging, inflammation, and melanogenesis-related genes in melanocytes treated with peptides and UV. Gene expression levels were normalized to GAPDH. Data are presented as mean ± SD (*n* = 4). “+” compared with the control group, “*” and the inhibition rates compared with the UV group, “#” compared with the SO1/SC1 combination group. ns = not significant, ^+/^*^/#^*P* < 0.05, ^++/**/##^*P* < 0.01, ^+++/***/###^*P* < 0.001.

To systematically investigate the molecular mechanisms underlying SEH’s anti-photoaging effects, we employed an integrated network pharmacology approach. RNA-seq analysis identified several major signaling pathways modulated by SEH treatment, including NF-κB, TNF, cell cycle, IL-17, phosphatidylinositol 3-kinase (PI3K)–Akt, cellular senescence, and MAPK signaling pathways. Based on our previous work, we selected 4 key receptors that are likely to mediate SEH’s bioactivity and compiled aging-related genes from public databases. Using STRING database analysis, we mapped molecular interactions among SEH-regulated pathways, target receptors, and aging-associated genes, generating a compound–target–pathway interaction network with 157 nodes and 1,300 edges, including 1,157 PPIs (Fig. 8A). The richness and connectivity of this network support a cooperative SPM model, in which multiple functional peptides within SEH act together through interconnected pathways to mitigate photoaging, rather than relying on a single dominant component.

Building on this network framework, we next focused on SO1 and SC1 as representative peptides within the SEH “mesh” and compared their effects under UV stress when used alone or in combination. At the transcriptional level, qualitative analysis revealed 3 distinct interaction patterns. The combination treatment demonstrated enhanced efficacy for *MMP3*, *IL-6*, and *MITF* compared to individual peptides (*P* < 0.05). For *P53*, *Collagen III*, *NFKB1*, *ERK*, *C-kit*, and *TYR*, the combination exhibited effects comparable to the single agents (*P* > 0.05). For *P16* and *P21*, the combined efficacy was lower than that of SO1 alone. Despite this variability, SO1 appeared to be the principal driver of anti-senescence and anti-inflammatory effects, whereas SC1 preferentially influenced melanogenesis-related pathways. Consequently, their combined use provided a more integrated regulation of UV-induced photoaging phenotypes than either peptide alone. As a functional “module” within the SEH network, the SO1/SC1 pair thus provides preliminary evidence that the anti-photoaging activity of SEH emerges from the collective action of multiple bioactive peptides, consistent with the proposed SPM mechanism (Fig. 8B).

**Fig. 9.**
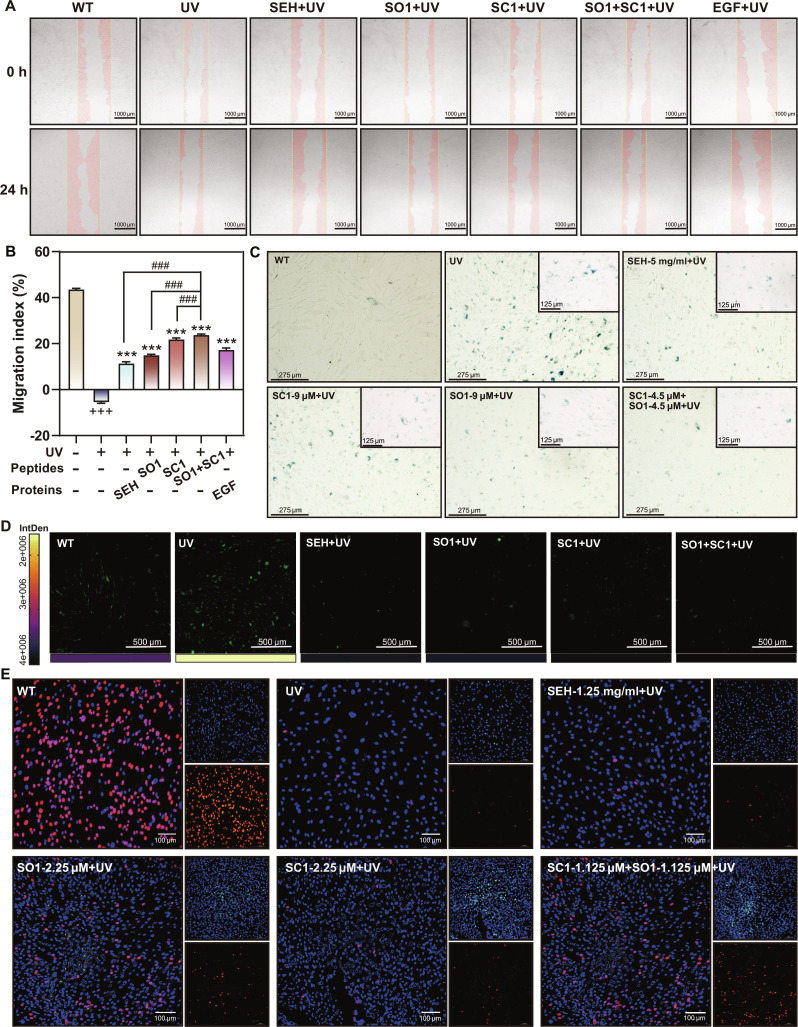
SO1 and SC1 peptides in combination exert comprehensive anti-photoaging effects. (A) Effects of different peptides on fibroblast migration following UV irradiation. Scale bar, 1,000 μm. (B) Quantitative analysis of cell migration rates. Effects of different peptides on fibroblasts. “+” compared with the control group, “*” compared with the UV group, “#” compared with the SO1/SC1 combination group. ^+++/***/###^*P* < 0.001. (C) β-Galactosidase staining. Scale bar, 275 μm. (D) ROS levels in fibroblasts under UV and peptide treatments. Scale bar, 500 μm. The color bar and IntDen values indicate the intensity of the ROS signal. (E) EdU staining under UV irradiation and peptide treatments. Scale bar, 100 μm. Insets show separated channels.

To assess the pro-migration effects of these peptides against UV-induced damage, we performed wound healing assays. While all treatments improved cell migration after UV exposure, the combination of SO1 and SC1 (1.25 μM each) demonstrated the strongest effect. Notably, this combination outperformed either peptide alone at a higher concentration (2.5 μM) and was superior to both the initial SEH extract and the EGF positive control (Figs. 9A and B). Consistent with these findings, we further examined multiple hallmarks of photoaging, including senescence-associated β-galactosidase activity, intracellular ROS levels, and proliferative capacity. The SO1/SC1 combination most effectively reduced SA-β-gal-positive staining, indicating a more pronounced attenuation of cellular senescence compared with single-peptide treatments (Fig. 9C). In parallel, all peptide groups decreased UV-induced ROS accumulation, but the combined SO1 and SC1 treatment produced the greatest reduction in ROS signal intensity (Fig. 9D). Moreover, EdU incorporation assays showed that the combination more robustly restored fibroblast proliferative activity under UV stress than either peptide alone, suggesting better recovery of cell renewal potential (Fig. 9E and Fig. S12).

SEH extract similarly promoted proliferation, albeit with reduced potency compared to the optimized peptide combination. These observations suggest that the relatively lower concentrations of individual bioactive peptides within SEH may attenuate its efficacy in specific cellular assays, despite its well-documented broad-spectrum anti-photoaging activities. Enrichment or supplementation of representative core peptides such as SO1 and SC1 could substantially enhance the therapeutic performance of SEH-based formulations, providing a rational strategy for optimizing silk protein-derived products in clinical applications.

Taken together, these results indicate that SO1 and SC1, as core components within the proposed SPM, cooperate to deliver a more comprehensive anti-photoaging profile. They can simultaneously promote cell migration and proliferation, alleviating senescence and suppressing oxidative stress. Collectively, these data demonstrate that SO1 and SC1 act as core components of the SPM, whose combined application exerts a synergistic and comprehensive anti-photoaging effect, thereby providing a solid foundation for subsequent human efficacy studies and potential applications.

### Clinical evaluation of the dual peptide system in reducing skin photoaging and restoring barrier effects in a human clinical trial

**Fig. 10.**
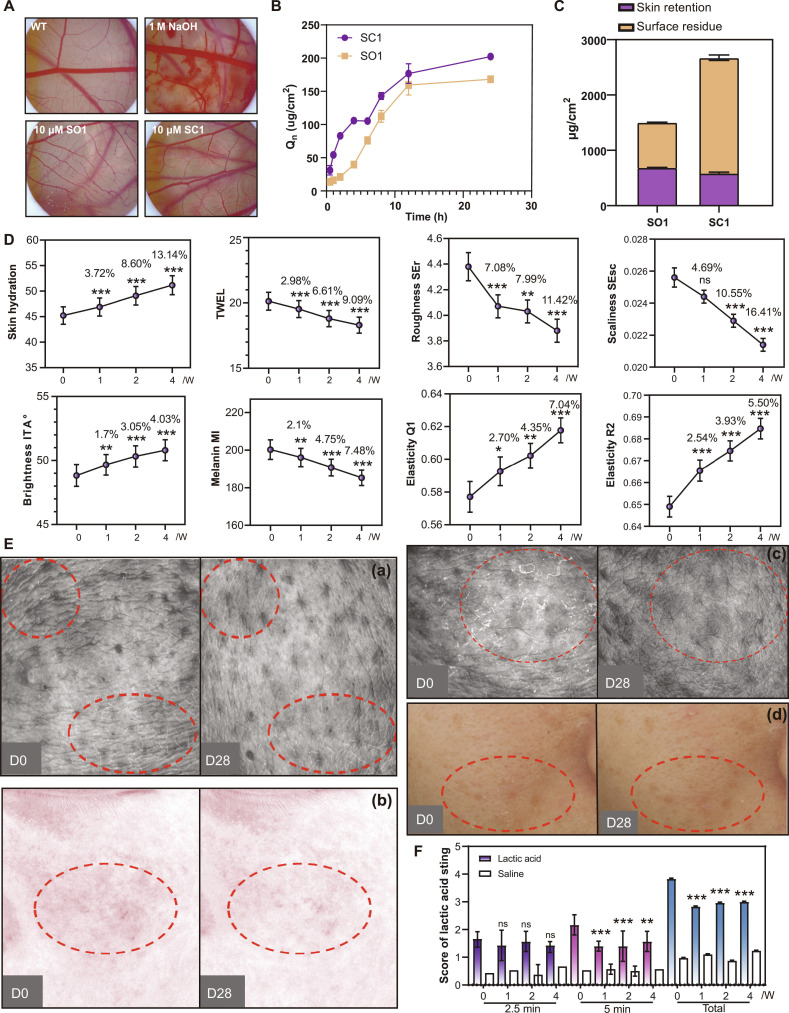
Results of clinical tests. (A) CAM characteristics in different treatments. (B) Cumulative permeation profiles showing time-dependent permeation amount (Qn, μg/cm^2^) through porcine skin over 24 h. Data are presented as mean ± SD (*n* = 6). (C) Mass balance analysis at 24 h showing skin retention (purple) and surface residue (orange) for both peptides. (D) Changes in skin parameters in 4 weeks. Data are presented as mean ± SD (*n* = 30). “*” and the rates of change compared with the baseline. ns = not significant, *P < 0.05, **P < 0.01, ***P < 0.001. (E) Comparison of skin characteristics at baseline (D0) and after 28 d (D28) using a skin stereomicroscope, with images (a) showing changes in skin roughness, (b) depicting barrier repair results, and (c) and (d) representing scaling and flakiness. (F) Scores of lactic acid sting effects measured over 4 weeks. Data are presented as mean ± SD (*n* = 30). “*” and the rates of change compared with the baseline. ns = not significant, ***P < 0.001.

To assess the safety profile of these peptides before further investigation, we conducted chick embryo chorioallantoic membrane (CAM) assays. Treatment with 10 μM of SEH, SO1, or SC1 showed no evidence of vascular irritation or toxicity compared to the untreated control, while the positive control (1 M NaOH) induced marked vascular damage (Fig. 10A and Fig. S13).

Furthermore, the patch test comprehensively evaluated the skin safety of SO1 and SC1 combinations. All 30 subjects tested at patch removal time points of 0.5, 24, and 48 h demonstrated identical results to the negative control, with no instances of erythema, swelling, edema, papules, or vesicles observed. These findings confirmed that the peptides were well-tolerated for topical application without showing skin safety concerns (Table 1).

**Table 1.** Patch test removal results

Sample name	Negative reaction (0)	Suspicious reaction; only slight erythema (1)	Weak positive reaction (erythema reaction); erythema, swelling, edema, possible papules (2)	Strong positive reaction (erythema reaction); erythema, swelling, edema, possible papules; reaction may extend beyond test area (3)	Very strong positive reaction (erythema reaction); obvious erythema, severe swelling, edema, confluent vesicles; reaction extends beyond test area (4)
Negative control	30	0	0	0	0
Test for 0.5 h	30	0	0	0	0
Test for 24 h	30	0	0	0	0
Test for 48 h	30	0	0	0	0

To evaluate the transdermal delivery potential of SC1 and SO1 peptides, in vitro skin permeation studies were performed using Franz diffusion cells with porcine skin over a 24-h period (Fig. 10B). The cumulative permeation amount (Qn) of both peptides increased progressively over time, exhibiting 3 distinct phases: an initial slow permeation phase (0 to 2 h), a steady-state permeation phase (2 to 8 h), and a plateau phase (>8 h). At 24 h, SC1 achieved a cumulative permeation of 202.33 ± 0.85 μg/cm^2^, approximately 20% higher than SO1 (168.27 ± 3.56 μg/cm^2^, mean ± SD, *n* = 6). Analysis of the steady-state permeation kinetics revealed distinct lag times (tlag) for the 2 peptides: SO1 exhibited a significantly shorter lag time (0.13 h) compared to SC1 (3.92 h) (Fig. S14), suggesting faster initial penetration through the stratum corneum barrier. Mass balance analysis at the end of the permeation study revealed that the majority of both peptides remained as surface residue, with SC1 showing substantially higher surface retention (approximately 2,085.82 ± 41.21 μg/cm^2^) compared to SO1 (approximately 815.05 ± 4.08 μg/cm^2^) (Fig. 10C). In contrast, the amount retained within the skin tissue was comparable between the 2 peptides: 587.25 ± 15.15 μg/cm^2^ for SC1 and 687.83 ± 3.79 μg/cm^2^ for SO1. These findings demonstrate that both peptides possess favorable transdermal penetration properties, with SC1 exhibiting superior permeation efficiency, while SO1 shows faster initial penetration and lower surface residue, supporting their potential for topical skincare applications.

Given that photoaging demonstrates as barrier dysfunction, texture deterioration, hyperpigmentation, and loss of elasticity, a clinical trial targeting female skin to address photoaging concerns in this population was conducted with the SO1/SC1 combination cream (Table S8). Based on the International Contact Dermatitis Research Group (ICDRG) grading criteria (Table S9), all 30 participants showed negative responses across all assessed adverse event categories (Table S10), confirming the favorable cutaneous safety profile of the SO1 and SC1 dual peptide system. The 28-d treatment led to significant improvements in photoaged skin, enhancing barrier function, hydration, and reducing water loss. It also improved skin texture by decreasing roughness and scaliness, addressed hyperpigmentation through increased brightness and decreased melanin levels, and enhanced skin elasticity, indicating improved dermal integrity. Notably, parameters such as transepidermal water loss (TEWL) and erythema typically increase when skin is exposed to irritating substances or experiences barrier disruption. The stability of these parameters throughout the 28-d application period provides additional evidence supporting the biocompatibility and stability of the SO1 and SC1 peptide combination during prolonged topical use (Fig. 10D and E). Besides, increased skin sensitivity is also a consequence of barrier dysfunction associated with photoaging. The peptide treatment improved this sensitivity. Lactic acid stinging tests showed reduced stinging responses after treatment (Fig. 10F). This indicated that sustained peptide treatment restored skin tolerance, suggesting improved barrier function.

## Discussion

**Fig. 11.**
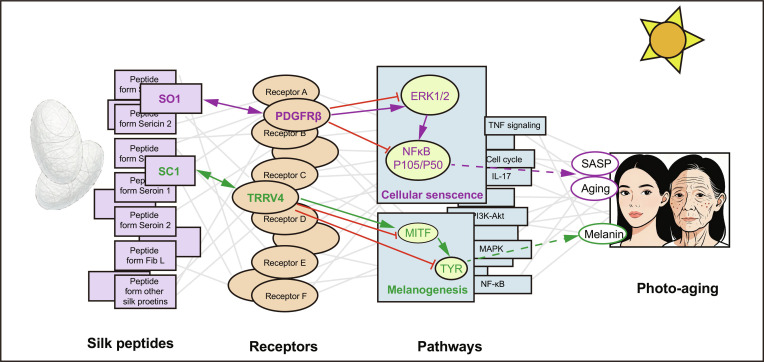
Schematic diagram of “Silk Peptides Mesh”.

This study revealed the important role of silk peptides in reducing photoaging, mainly by adjusting the senescence pathways in melanocytes exposed to UV radiation, while also universally benefiting keratinocytes and fibroblasts. We discovered 2 peptides that showed the primary biological effects. SO1 inhibited photoaging through the PDGFRβ/ERK/NF-κB pathway, and SC1 reduced melanin synthesis via the TRPV4/MITF pathway. To systematically elucidate the anti-aging mechanisms of silk, we integrated multi-omics with network pharmacology to move beyond a single-molecule analysis, defining the SPM. The efficacy of SO1 and SC1 was subsequently confirmed in clinical trials, validating the broader therapeutic potential of SEH-derived peptides (Fig. 11).

While our findings demonstrate the clinical effect of SO1 and SC1, the current study adopted a longitudinal self-controlled design to capture dynamic skin improvements, rather than conducting a double-blind, placebo-controlled trial comparing directly against standard agents like retinoids. Additionally, the relatively small and female-dominant cohort may limit the generalization of our results. However, considering the conservation of the identified biological pathways, these benefits likely reflect an intrinsic therapeutic potential. Future studies with larger, sex-balanced cohorts are warranted to validate and extend the present findings.

For the silkworm, the cocoon acts as a dual-defense system, integrating fibroin’s physical strength with sericin’s biological protection. The inner fibroin core provides structural toughness [15], consistent with the low bioactivity and immunogenicity observed in our docking analysis. Conversely, the outer sericin coating functions not only as a structural binder but also as a bioactive shield, actively defending the pupa against environmental threats [25]. It is this bioactive-rich outer layer that gives rise to SO1 (derived from Seroin-1) and SC1 (derived from Sericin-1) identified in this study.

However, this evolutionary design implies inherent complexity. In silk cocoon, SO1 and SC1 coexist with other proteins and peptides, forming a collective bioactive shield. Although limited by lower individual component abundance, SEH’s multi-peptide network enables multi-dimensional regulation, explaining its widely reported diverse biological activities. Based on this systemic perspective, we integrated multi-omics with network pharmacology to define the SPM. This approach moves beyond the limitations of single-molecule analysis, elucidating how 105 distinct peptides interact with receptors and modulate signaling pathways to improve downstream clinical indicators, including anti-inflammatory, anti-melanogenic, and anti-aging effects. Just as this invisible mesh provides comprehensive biochemical protection for the pupa during metamorphosis, its constituent peptides can now offer a similar systemic protective function for human skin.

Taken together, the SPM provides a theoretical framework for the broad efficacy of silk-derived peptides, wherein SO1 and SC1 retain distinct regulatory functions. Crucially, while our results revealed differential gene expression profiles for the SO1/SC1 combination, their cotreatment ultimately elicited superior phenotypic protection against UV-induced photoaging. Furthermore, preliminary molecular docking suggests that other peptides within the network also engage shared or related targets, implying a landscape of complexity among the 105 peptides. Defining these interactions precisely will necessitate incorporating expanded datasets of single and combined novel peptides into rigorous quantitative pharmacological models, such as the Chou–Talalay method, in future studies.

From a practical standpoint, this “Mesh” serves as a foundational platform for our future study. Beyond identifying single potent peptides, this network enables us to map upstream and downstream targets aligned with clinical demands, facilitating the strategic selection of multiple active peptides. This combinatorial approach achieves a comprehensive therapeutic effect, thereby amplifying potential and broadening the scope of treatment. Ultimately, this conceptual shift from single molecules to an assembled functional network will guide our continued exploration of the clinical applications of silk peptides.

## Materials and Methods

### Cell lines and chemicals

Silkworm cocoons were practical varieties purchased from Chongqing Sericulture Science and Technology Research Institute. Human primary melanocytes and fibroblasts were obtained with the culture medium from BIOCELL, China. Pig1 human melanocytes were generously provided by H. He of Southwest University. HaCaT keratinocytes and B16F10 murine melanoma cells were stored in our laboratory. Dulbecco’s modified Eagle’s medium (DMEM) and fetal bovine serum (FBS) were from Gibco (USA). Milli-Q water was used in all experiments. All other chemicals were of analytical grade or better.

### Silk protein extraction and hydrolysis

The initial material of the silkworm cocoon was washed 3 times with pure water and cut into small pieces of approximately 1 cm^2^. The pieces were then extracted and hydrolyzed as described in Table S1. After extraction and hydrolysis, the mixture was filtered to obtain SEH, and finally freeze-dried for storage.

### Characterization of silk protein extraction samples

A scanning electron microscope (Hitachi, Japan, SU3500) was used to examine the silk structure after sericin extraction. Sericin concentration was measured following a bicinchoninic acid assay (BCA) kit (Beyotime, China, P0012). The molecular weight of sericin was identified with SDS-PAGE assay. Sericin samples with the same solid content were prepared, and loading buffers were added and boiled for 5 min. Electrophoresis was performed on a 12% gel, which was then stained with Coomassie Blue Dye and scanned with a Patch clamp system (Canon, Japan). The silk degumming rate was calculated by subtracting the weight of degummed silk cocoons from the initial weight of dry silk cocoons and dividing by the initial weight.

### Cell culture

Cells were cultured in a 37 °C incubator with 5% CO_2_ with culture dishes (Corning, USA) and supplemented with 10% FBS (Gibco, USA). All operations were conducted following strict aseptic techniques.

### Cell melanin content assay

Melanin content in cells is expressed as the amount of melanin produced per unit of protein. B16F10 cells were treated with active substances at varying concentrations for 12 h. Half of the cells were solubilized in 1 M NaOH at 80 °C for 1 h, and absorbance at 405 nm was measured using a microplate reader (Synergy H4, USA). The protein concentration of the other half was determined using a BCA assay. Semiquantitative melanin content was calculated by dividing the melanin absorbance by the protein concentration.

### Cell viability

Logarithmic phase cells were inoculated in 96-well plates until reaching 80% confluence. The cells were treated with sericin samples for 24 h and then incubated with Cell Counting Kit-8 (CCK-8) (Yeasen, China) for 30 min. Absorbance was measured at 450 nm using a microplate reader to calculate cell viability.

### Endotoxin quantification

Endotoxin content was measured using a chromogenic Limulus Amebocyte Lysate (LAL) assay kit (GenScript, L00350) according to the manufacturer’s instructions. Briefly, standard endotoxin solutions were prepared by serial dilution to generate a concentration range of 0.01 to 1.0 EU/ml. In a 96-well microplate, 50 μl of each standard or appropriately diluted sample was added in triplicate, followed by the addition of LAL reagent and chromogenic substrate. After incubation and reaction termination, absorbance was measured at 545 nm using a microplate reader. A standard curve was constructed by plotting absorbance values against endotoxin concentrations, and sample endotoxin levels were calculated by interpolation and expressed as endotoxin units per milliliter (EU/ml).

### UV irradiation

After 6 h of pretreatment of SEH, peptides, or other active ingredients, the cell culture medium was discarded and 1 ml of phosphate-buffered saline (PBS) was added. All groups except the blank control received UV irradiation at a dose of UVA 75 mJ/cm^2^ + UVB 75 mJ/cm^2^. After exposure, PBS was replaced with fresh culture medium and cells were incubated at 37 °C with 5% CO_2_ for 6 h. Control groups were treated identically without UV exposure. Cells were prepared for subsequent tests.

UVA and UVB irradiation were delivered using a UV irradiation system (HOPE-MED 8134C, Tianjin, China). The irradiance intensity was calibrated using a UV radiometer (Lutron Electronic Enterprise UV-340A, Taiwan, China). Cells were exposed to 75 mJ/cm^2^ UVA and 75 mJ/cm^2^ UVB sequentially, with exposure time calculated based on the measured irradiance.

### RNA-seq and analysis

Transcriptome sequencing was performed on 3 cell groups, including wild-type (WT) controls, 1mg/ml SEH pretreated with UV, and UV-only treated cells, with a dose of UVA 75 mJ/cm^2^ + UVB 75 mJ/cm^2^. Total RNA was extracted, and RNA sequencing libraries were constructed using Illumina technology. Differentially expressed genes were identified using DESeq2 [26], followed by pathway enrichment analyses using the gseGO and gseKEGG parameters from the clusterProfiler package [27]. Boxplots were created with the ggplot2 package, ternary plots were created with ggtern (V3.5.0), and calculations were conducted on a high-performance computer (Sugon, China, I950rG).

### TYR inhibition kinetic assay

TYR activity was assessed using tyrosine and L-3,4-dihydroxyphenylalanine (L-DOPA) as substrates. Reactions were carried out in a 96-well microplate, with each well containing 100 μl of 0.1 M phosphate buffer (pH 6.8), 20 μl of TYR enzyme solution, and 20 μl of sericin. After preincubating the mixture for 10 min at 37 °C, 20 μl of tyrosine and L-DOPA was added to initiate the reaction. The formation of dopachrome was monitored by measuring absorbance at 475 nm every minute for 30 min. Reactions were also conducted with varying L-DOPA concentrations, and kinetic parameters (*V*_max_ and *K*_m_) were determined using the Michaelis–Menten equation [28].

### Copper ion chelation assay

Different concentrations of SEH solution were prepared, along with treatment groups including SEH + CuSO_4_, SEH + TYR, and a PBS control. Absorbance measurements were performed using a microplate reader (Synergy H4, USA) over a wavelength range of 200 to 800 nm. The absorbance values for each treatment group were recorded at various wavelengths.

### TYR conformational changes

Conformational changes in TYR were assessed by circular dichroism (CD) spectroscopy. Spectra for TYR alone, SEH alone, and a mixture of TYR and SEH were recorded from 190 to 260 nm at room temperature. Each sample was prepared in PBS (pH 6.8) and analyzed in a 1.0-mm quartz cuvette.

### qRT-PCR analysis

The total mRNA of cells was isolated by Trizol (Invitrogen. USA) and reversely transcribed to complementary DNA (cDNA) at 42 °C (E047-01B), and NovoStart SYBR qPCR SuperMix plus (E096) was used as the fluorescent dye (Novoprotein, China). The relative mRNA level was normalized with glyceraldehyde-3-phosphate dehydrogenase (GAPDH). DNA sequence synthesis was completed in BGI Genomics, China. Primers were shown in Table S11.

### Liquid chromatography–tandem mass spectrometry

SEHs were desalted and concentrated using C18 solid-phase extraction columns. Peptides were analyzed by LC-MS/MS on a high-resolution mass spectrometer (Thermo Fisher Scientific, Germany, Q-Exactive). Parameters included a precursor ion scan range of mass/charge ratio (*m/z*) 350 to 1,550 and a fragment ion scan range starting from *m/z* 110. Data-dependent acquisition (DDA) used a top 20 method with dynamic exclusion, selecting peptides for fragmentation based on intensity. The resulting spectra were matched against the silkDB database using SequestHT to identify peptide sequences.

### Construction of core gene PPI subnetwork

The aging gene set was confirmed in the OMIM and GeneCards databases using the keywords “aging” and “senescence”, followed by generating a Venn diagram using the VennDiagram package to visualize the overlap with KEGG-enriched genes. Constructing a preliminary PPI subnetwork focused on hub genes from the overlapping set. These genes were mapped to STRING protein IDs using the 9606.protein.aliases.v12.0.txt file, prioritizing official naming sources (BioMart_HUGO, Ensembl_HGNC, and HGNC). High-confidence PPIs were extracted from the 9606.protein.links.v12.0.txt file with a filtering criterion of a combined score ≥ 400. The resulting nodes were visualized using Cytoscape (V3.9.1).

### Molecular dynamics simulation

A full-atom molecular dynamics simulation was performed using AMBER 22 [29]. Peptide charges were calculated using the antechamber module and Gaussian 09 software’s Hartree–Fock (HF) SCF/6-31G* method. The crystal structures of target receptors were obtained from the Protein Data Bank (PDB) with the following accession codes: TRPV4 (7M97), IGF1R (5FXS), PDGFRβ (5GRN), MC1R (7F4D), MITF (6G9F), TYR (5M8M), TRP1 (5M8O), and TRP2 (5M8P). The peptide and protein were described with the GAFF2 and ff14SB force fields, respectively. Hydrogen atoms were added, and a truncated octahedral TIP3P solvent box was inserted at a 10-Å distance [30], followed by the addition of Na^+^/Cl^−^ ions to balance the charge. Topology and parameter files for simulation were generated.

System energy was optimized with 2,500 steps of steepest descent and 2,500 steps of conjugate gradient methods. The system was then heated for 200 ps from 0 K to 298.15 K, followed by a 500-ps constant number of particles, volume, and temperature (NVT) ensemble simulation at 298.15 K to uniformly distribute solvent molecules. A 500-ps constant number of particles, pressure, and temperature (NPT) equilibration simulation was conducted, leading to a final 100-ns NPT ensemble simulation under periodic boundary conditions. The nonbonded cutoff distance was set to 10 Å, and the particle mesh Ewald (PME) method was used for long-range electrostatics [31]. The SHAKE method constrained hydrogen bonds, the Langevin algorithm controlled temperature (γ = 2 ps^−1^), system pressure was maintained at 1 atm, and trajectories were saved every 10 ps for analysis.

### MM/GBSA binding free energy calculation

The binding free energy between proteins and ligands for all systems was calculated using the MM/GBSA method [32–34]. In this study, MD trajectories from 90 to 100 ns were used for calculations, with the specific formula as follows:

$$\begin{array}{c} \begin{array}{cc} {\Delta G}_{\text{bind}}\; & \;={\Delta G}_{\text{complex}}-\left({\Delta G}_{\text{receptor}}+{\Delta G}_{\text{ligand}}\right)\\ & ={\Delta E}_{\text{internal}}+{\Delta E}_{\mathrm{VDW}}+{\Delta E}_{\text{elec}}+\Delta G_{\mathrm{GB}}+{\Delta G}_{\mathrm{SA}} \end{array} \end{array}$$ (1)

In Eq. (1), Δ*E*_internal_ represents internal energy, Δ*E*_VDW_ represents van der Waals interactions, and Δ*E*_elec_ represents electrostatic interactions. Among these, internal energy includes bond energy (*E*_bond_), angle energy (*E*_angle_), and torsion energy (*E*_torsion_). Δ*G*_GB_ and Δ*G*_GA_ collectively refer to solvation free energy. Specifically, GGB represents polar solvation free energy, and GSA represents nonpolar solvation free energy. For Δ*G*_GB_, this study adopts the GB model with igb = 2 [35]. Nonpolar solvation free energy (GSA) is calculated based on the product of surface tension (γ) and solvent-accessible surface area (SA), where GSA = 0.0072 × SASA. Entropy change was neglected due to high computational resource requirements and low precision [32].

### MST assay

MST experiments were performed to determine the binding affinity between peptides and target proteins using a Monolith NT.115 instrument (NanoTemper Technologies). Target proteins were fluorescently labeled according to the manufacturer’s protocol. A series of 16 serial dilutions of the peptide ligands were prepared in MST buffer. Each ligand dilution was mixed with an equal volume of labeled protein at a constant concentration and incubated at room temperature for 30 min to reach binding equilibrium. The samples were loaded into standard capillaries and measured using medium MST power. The change in normalized fluorescence (ΔFNorm [%]) was plotted against ligand concentration, and the dissociation constant (*K*_d_) was calculated by fitting the data to a dose–response curve using MO.Affinity Analysis software.

### ROS assay

Cells were pretreated with protein or peptides, and the supernatant was discard. The cells were exposed to UV radiation (UVA 75 mJ/cm^2^ + UVB 75 mJ/cm^2^), followed by subsequent incubation. The cell culture medium was removed, and 1 ml of the ROS probe was added to each well. The cells were incubated in a shaking CO₂ incubator at 37 °C with 5% CO₂ for 30 min. After the ROS detection solution was discarded, the cells were washed 3 times with basal culture medium. Finally, 1 ml of basal culture medium was added, and microscope images were taken using a microscope (DMi8 Leica, Germany).

### β-Galactosidase staining assay

The HaCaT cells were seeded into a 6-well plates and incubated overnight in a CO₂ incubator. The cells were pretreated with active protein or peptides, the supernatant was discarded, and the cells were exposed to UV radiation at a dose of UVA 75 mJ/cm^2^ + UVB 75 mJ/cm^2^. After incubation, the cell culture medium was removed. Each well was washed with 1 ml of PBS, and 1 ml of β-galactosidase fixing solution was then added for fixation at room temperature for 15 min. The fixing solution was discarded, and the cells were washed 3 times with PBS. The staining solution was added, and the cells were incubated in a CO₂-free incubator at 37 °C for about 18 h. Finally, the staining solution was discarded, 1 ml of PBS was added, and images were captured under a microscope (DMi8 Leica, Germany).

### Western blot

The cells were pretreated with peptides, exposed to UV radiation, and then incubated for 6 h. The cells were washed twice with cold PBS to remove any residual media or treatments. Protein extraction was carried out by adding lysis buffer containing protease and phosphatase inhibitors, after which the cells were scraped and incubated on ice for 30 min. The cell lysate was then centrifuged at 12,000 rpm for 10 min at 4 °C, and the supernatant was collected. Protein concentration was determined using a BCA assay. Equal amounts of protein were mixed with loading buffer, heated at 95 °C for 5 min, and loaded onto an SDS-PAGE gel. The proteins were transferred to a polyvinylidene difluoride or nitrocellulose membrane, which was subsequently blocked with 5% nonfat milk or bovine serum albumin in tris-buffered saline with Tween 20 (TBST) for 1 h at room temperature. The membrane was incubated overnight at 4 °C with primary antibodies, followed by washing with TBST and incubation with secondary antibodies for 1 h. Detection was achieved using enhanced chemiluminescence (ECL) substrate (Thermo Scientific, USA), and the membrane was imaged using a chemiluminescence imaging system, using ImageJ analysis quantification of bands.

### Transmission electron microscopy

The cells were pretreated with 2.25-μm SC1 peptides for 6 h, the supernatant was discarded, and the cells were then exposed to UV radiation followed by subsequent incubation. Cells were fixed for 2 h at 4 °C with 2.5% glutaraldehyde in 0.1 M phosphate buffer (pH 7.4). After fixing, they were treated with 1% osmium tetroxide in the same buffer for 1 h. The samples were dehydrated using a graded ethanol series and embedded in epoxy resin. Ultrathin sections (70 nm) were prepared with an ultramicrotome and placed on copper grids. The sections were stained with uranyl acetate and lead citrate for contrast enhancement. Electron micrographs were taken using a transmission electron microscope at 80 kV and were recorded digitally for further analysis.

### siRNA transfection

HaCaT cells or fibroblasts were seeded in 6-well plates at a density of 2 × 10^5^ cells per well and cultured overnight to reach approximately 60% to 70% confluence. Cells were then transfected with siRNA targeting TRPV4 for HaCaT cells or PDGFRβ (BGI Genomics, China) for fibroblasts using Lipofectamine RNAiMAX (Invitrogen, USA) according to the manufacturer’s instructions. Briefly, 50 pmol of siRNA was diluted in 125 μl of Opti-MEM medium, and 5 μl of Lipofectamine RNAiMAX was diluted in another 125 μl of Opti-MEM medium. After 5-min incubation at room temperature, the 2 solutions were mixed gently and incubated for 20 min to form siRNA–lipid complexes. The mixture was then added dropwise to the cells. A scrambled siRNA was used as a negative control. The siRNA sequences were as follows: siTRPV4 sense: 5′-GCAAUGAGGACCAGACCAA(dT)(dT)-3′, antisense: 5′-UUGGUCUCGGUCCUCAUUGC(dT)(dT)-3′; siPDGFRβ sense: 5′-UCUCUUCGAGAAGCAGCACC(dT)(dT)-3′, antisense: 5′-GGUGCUGCUUCUCGAGAGA(dT)(dT)-3′. Cells were incubated with the transfection mixture for 36 h before subsequent treatments and analyses. Knockdown efficiency was validated by qRT-PCR.

### Network pharmacology analysis

Using the STRING database, an interaction network was constructed between peptides in SEH and photo-aging. The peptides in SEH were linked to receptors based on molecular docking to form a network relationship. Subsequently, the receptors were correlated with key signaling pathways regulated by SEH based on STRING data, ultimately generating a network diagram of gene targets related to photo-aging.

### Cell scratch assay

The fibroblasts were seeded into a 6-well plate and incubated overnight. A sterile pipette tip was used to create uniform scratches across the monolayer in each well. After the scratches were made, the wells were gently washed with serum-free high-glucose DMEM to remove any cell debris, and then filled with treatment media. Treatments included SEH at 2.5 mg/ml, SO1 at 2.5 μM, SC1 at 2.5 μM, SO1 + SC1 at 1.25 μM + 1.25 μM, and EGF at 10 ng/ml in serum-free high-glucose DMEM. Microscope images were taken at 0 h and after 24 h (DMi8 Leica, Germany).

### Ocular irritant and corrosive hen’s egg test-chorioallantoic membrane (HET-CAM) test

SPF Bai Laihang chicken embryos weighing 50 to 60 g were incubated at 37.5 ± 0.5 °C with 55% to 70% humidity, turning the eggs 3 to 6 times per hour. On day 9, healthy embryos were selected. The air chamber was marked, and the area was removed to expose the white membrane, which was moistened with a 0.9% NaCl solution. After removing the inner membrane without damaging the CAM, 0.3 ml of SEH or peptides solution was applied. The membrane was incubated for 5 min before being rinsed with PBS. The CAM was then immediately observed and scored for signs of irritation at set time points.

### Patch test

The inner forearm skin of volunteers is tested. The substance to be tested is applied to the skin and covered with a breathable patch. Skin reactions, including redness and itching, are typically observed over 48 h. After the patch is removed, reactions are scored based on their severity to determine whether the tested substance causes an allergic reaction.

### In vitro skin permeation study

Fresh porcine skin from the dorsal and abdominal regions of 28- to 42-day-old Bama miniature pigs (thickness: 0.8 to 1 mm) was purchased and stored frozen until use. Prior to the experiment, the skin was thawed at room temperature, rinsed with physiological saline, and trimmed to remove any residual subcutaneous fat. The skin was then cut into circular pieces with a diameter of 20 mm. The Franz diffusion cell consisted of a donor chamber and a receptor chamber with an effective diffusion area of 1.76625 cm^2^.

The receptor chamber was filled with 14 ml of pH 7.4 PBS (filtered through a 0.22-μm membrane) and equipped with a magnetic stirrer. The prepared porcine skin samples were mounted between the donor and receptor chambers with the stratum corneum facing upward, secured using stainless steel clamps. Air bubbles were carefully removed by gently tapping the chamber wall.

The donor chamber was loaded with 0.5 ml of 2.4 mg/ml SC1 or 1.6 mg/ml SO1 solution and sealed with parafilm to prevent evaporation. The assembled Franz diffusion cells were maintained at 32 ± 0.5 °C in a water bath with continuous stirring at 300 rpm. Samples (1 ml) were withdrawn from the receptor chamber at 0.5, 1, 2, 4, 6, 8, 12, and 24 h and immediately replaced with an equal volume of fresh prewarmed PBS. The concentration of SC1 or SO1 in each sample was determined by high-performance liquid chromatography analysis. Each experiment was performed in triplicate.

The cumulative permeation amount per unit area ($Q_{n}$, μg/cm^2^) was calculated using the following equation:

$$Q_{n}=\frac{V\times C_{n}+\sum_{i=1}^{n-1}C_{i}\times V_{i}}{A}$$ (2)

where $Q_{n}$ is the cumulative permeation amount per unit area (μg/cm^2^), V is the volume of the receptor chamber (14 ml), $V_{i}$ is the volume of each withdrawn sample (1 ml), $C_{i}$ is the concentration in the *i*th sample (μg/ml), $C_{n}$ is the concentration in the *n*th sample (μg/ml), and A is the effective diffusion area (cm^2^).

### In vivo validation in human subjects

Two parts of the clinical skin experiments were conducted. The preliminary experiment was a double-blind, split-face pilot study involving 2 participants (one female, 24 years, and one male, 40 years) that evaluated the efficacy of 2 mg/ml SEH cream over a 4-week period, with the cream applied to one-half of the face and a control cream to the other half.

The formal experiment was a single-center, self-controlled, single-arm, open-label clinical trial involving 30 healthy Chinese women aged 18 to 55 years, focusing on newly developed natural peptides SO1 and SC1. Participants exhibited visible signs of photoaging, dry skin, and self-reported skin sensitivity. After obtaining written informed consent, they discontinued all other facial skincare products and exclusively applied the test cream twice daily for 28 d.

Clinical evaluations were conducted at baseline and at weeks 1, 2, and 4, following standardized facial cleansing and a 30-min acclimatization period in controlled conditions (20 to 22 °C, 40% to 60% humidity). Skin assessments utilized MPA580 to evaluate viscoelastic properties and other physiological parameters, while VC98 was employed to assess surface topography (Courage Khazaka Electronic, Germany). Additionally, facial imaging was performed using the VISIA-CR imaging system (Canfield Scientific, USA). Parameters such as skin spots and UV spots were outputs from the VISIA, MPA580, and VC98 system.

### Lactic acid sting test

A 10% lactic acid solution was applied to one nasolabial fold, with normal saline as a negative control. Stinging sensations were rated at 2.5 and 5 min using a 5-point scale, and cumulative scores compared lactic acid and saline responses to evaluate the anti-irritation effects of the peptide formulation. Additionally, participants conducted self-assessments of skin hydration, smoothness, brightness, and overall satisfaction at each visit, with scores of 3 and 4 deemed clinically effective responses for evaluating treatment efficacy.

### Statistical analysis

Data were presented as mean ± SD. Two-tailed Student’s *t* tests were performed for statistical analysis. “+” indicated increase, “*” indicated inhibition, and the numbers indicated the inhibition rates compared to the UV group. ns = not significant, ^+/^*P < 0.05, ^++/^**P < 0.01, ^+++/^***P < 0.001.

## Ethical Approval

This study was approved by the Ethics Committee of the Ninth People’s Hospital of Chongqing (approval no. 2021-002), and written informed consent was obtained from all participants.

## Data Availability

The data that support the findings of this study have been deposited into CNSA [36] with accession number CNP008231.
