# Supplementary material for: A Silk-Derived Dual Peptide System Suppresses Skin Photoaging by Inhibiting PDGFRβ-Mediated Cellular Senescence and TRPV4-Mediated Melanogenesis
Source: Research (Wash D C). 2026 May 8;9:1255. doi: 10.34133/research.1255 (PMC13153459; doi:10.34133/research.1255)
Supplement: Supplementary 1 — Figs. S1 to S14 Tables S1 to S11 [file research.1255.f1.zip › research.1255.f1.docx]

SUPPLEMENTARY MATERIALS


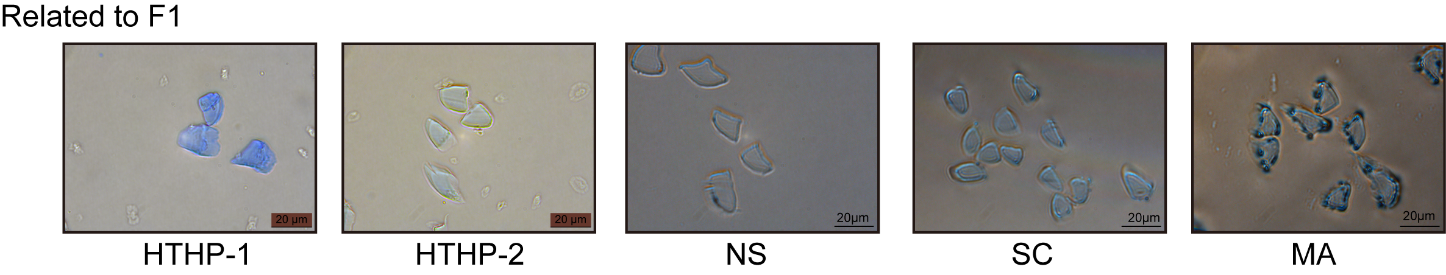


***Fig. S1.*** *Cross-sectional electron microscope image of silk fibers after degumming.*


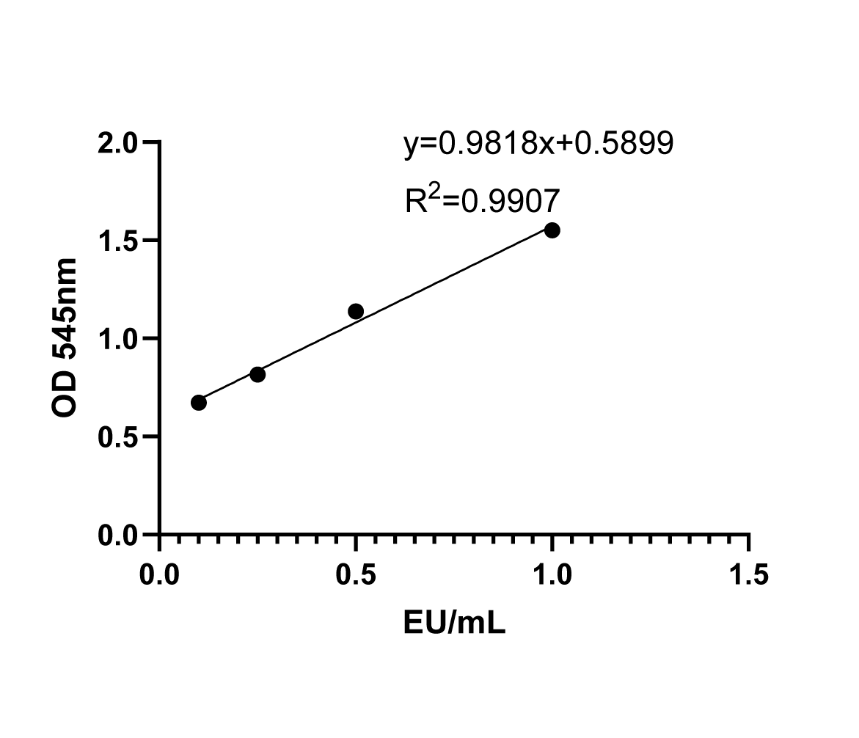


***Fig. S2.*** *Standard curve for testing endotoxin.*


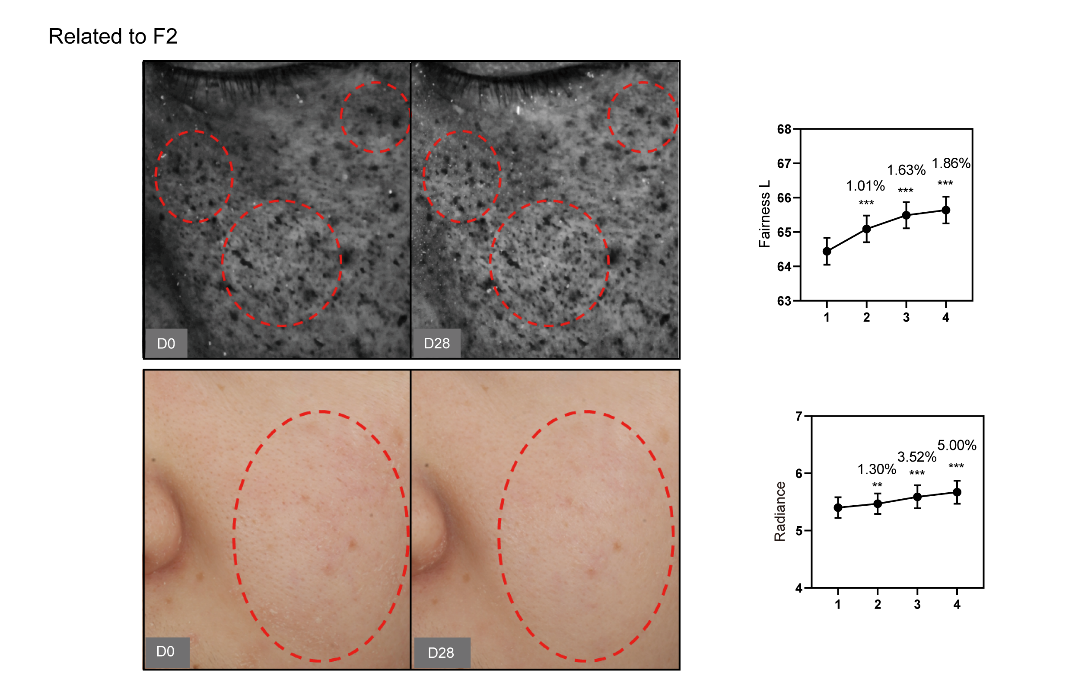


***Fig. S3.*** *Comparison of skin characteristics at baseline (D0) and after 28 days (D28).*


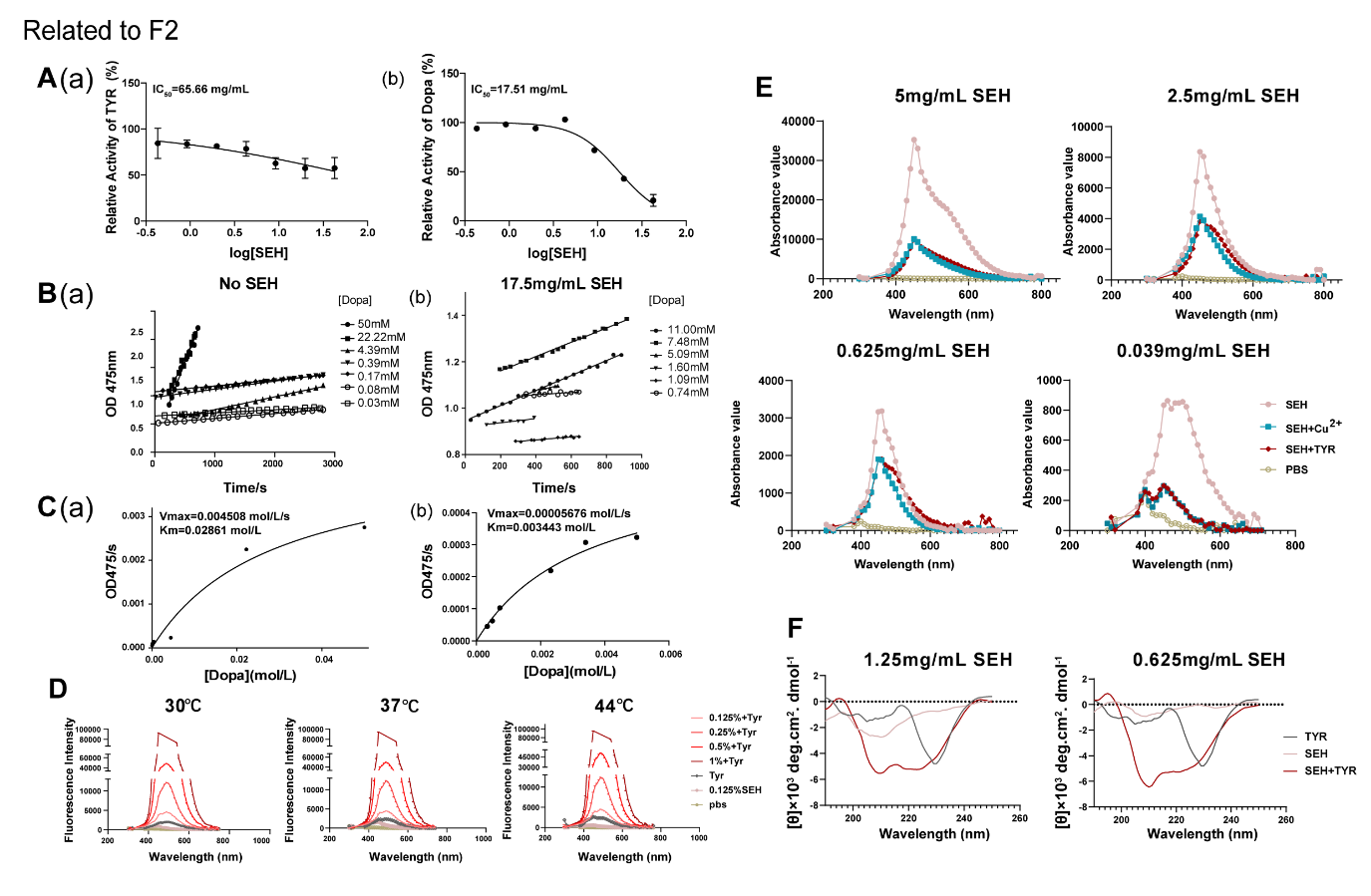


***Fig. S4.*** *Interactions of SEH with Tyrosinase. (A) Inhibition assays for TYR and Dopa using varying concentrations of SEH. (B) Kinetic analysis of Dopa conversion measured over time at different concentrations (a) without SEH and (b) with 17.5mg/mL SEH. (C) Michaelis-Menten kinetics for Dopa concentrations, with fitted curves indicating Vmax and Km values for (a) no SEH and (b) in the presence of 17.5 mg/mL SEH. (D) Fluorescence intensity profiles for tyrosinase and varying concentrations of SEH at temperatures of 30°C, 37°C, and 44°C. (E) UV-Vis absorbance spectra for various concentrations of SEH interactions with Cu² and TYR. (F) Circular dichroism spectra for 1.25 mg/mL and 0.625 mg/mL SEH combined with TYR.*


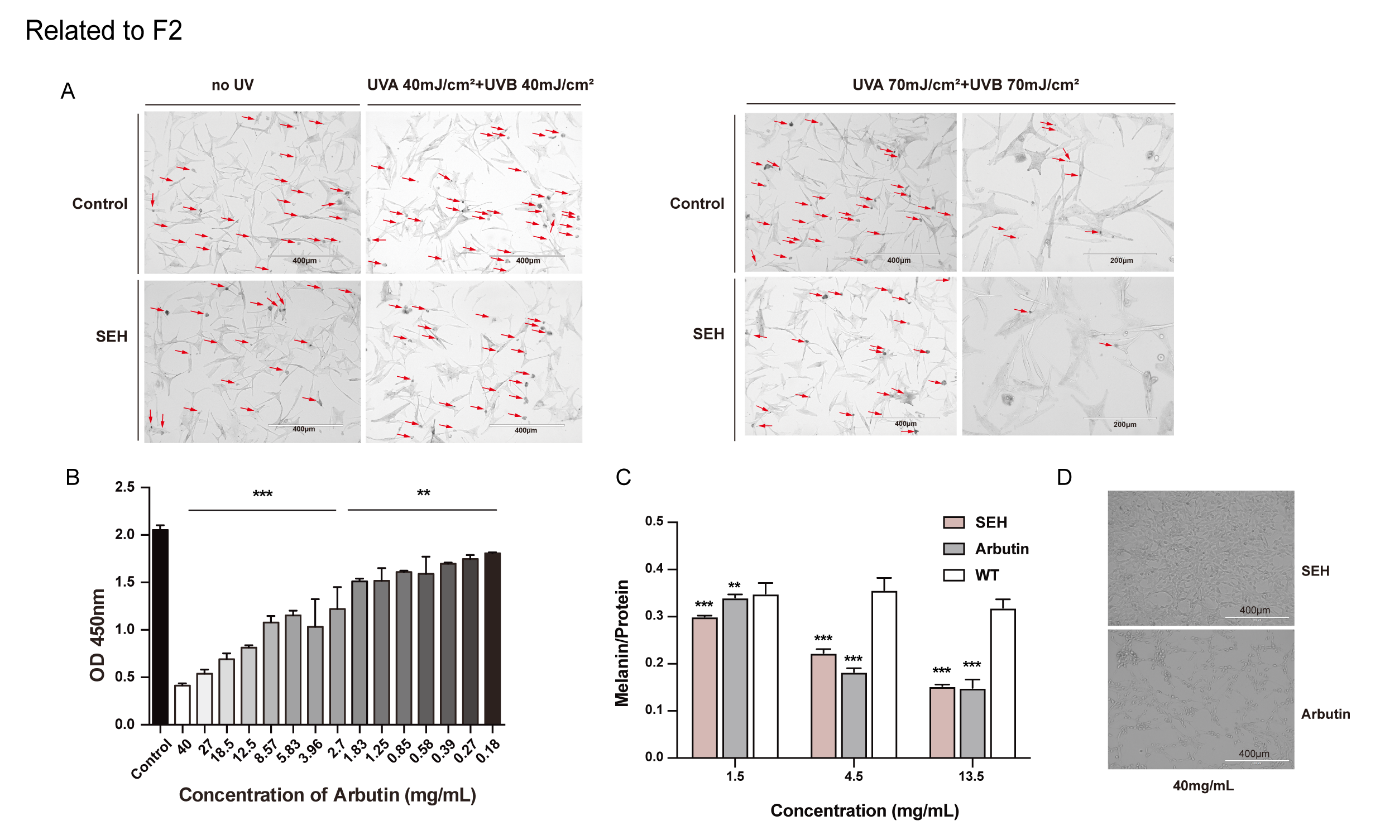


***Fig. S5.*** *Effects of SEH and Arbutin on cellular changes following UVB exposure. (A) Microscopic images of primary human melanocytes morphology in control and SEH-treated groups under different UVB irradiation intensities . Red arrows indicated changes in cell morphology. (B) Cell viability measured by OD 450 nm readings after treatment with varying concentrations of Arbutin (0.18–40 mg/mL), reflecting absorbance across different concentrations. (C) Bar graph displaying melanin content relative to protein concentration in Arbutin and SEH treatment groups at concentrations of 1.5, 4.5, and 13.5 mg/mL compared to the control group. (D) Microscopic images showing changes in cell morphology for the 40 mg/mL SEH and Arbutin treatment groups.*


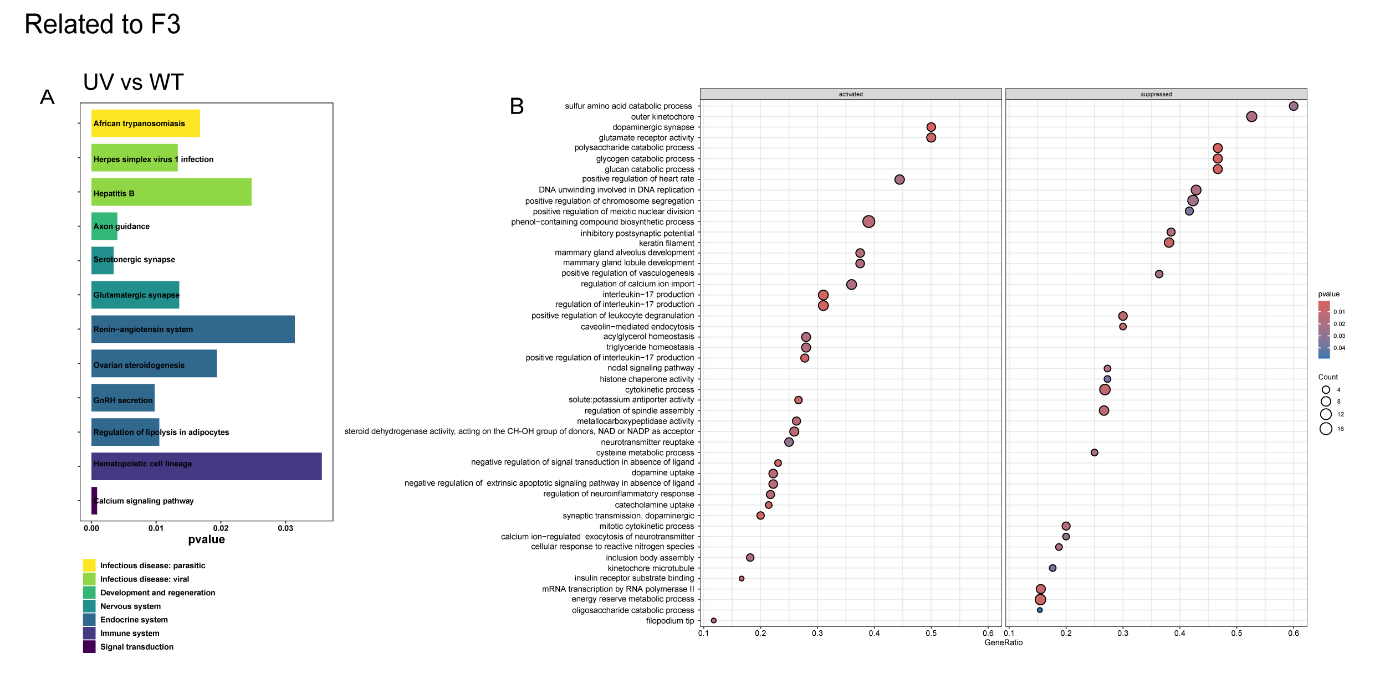


***Fig. S6.*** *Gene Set Enrichment Analysis (GSEA) of UV vs. WT group. (A) Enriched pathways associated with UV exposure compared to WT. (B) Pathways activated (left) and suppressed (right) following UV exposure, with points representing pathways and colors indicating statistical significance.*


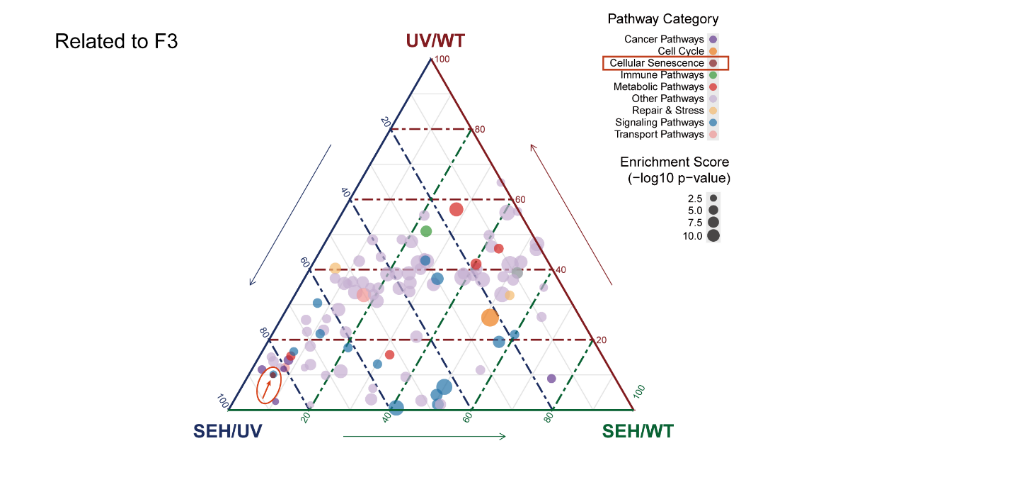


***Fig. S7.*** *Ternary plot visualizing and comparing the enrichment of KEGG pathways across UV/WT, SEH/UV, and SEH/WT group.*


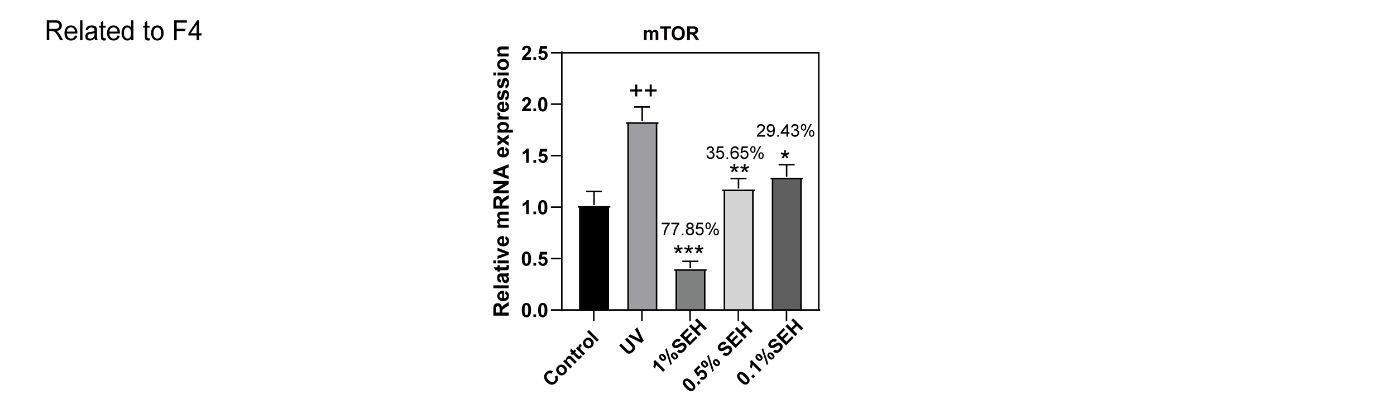


***Fig. S8.*** *qPCR analysis of mTOR.*


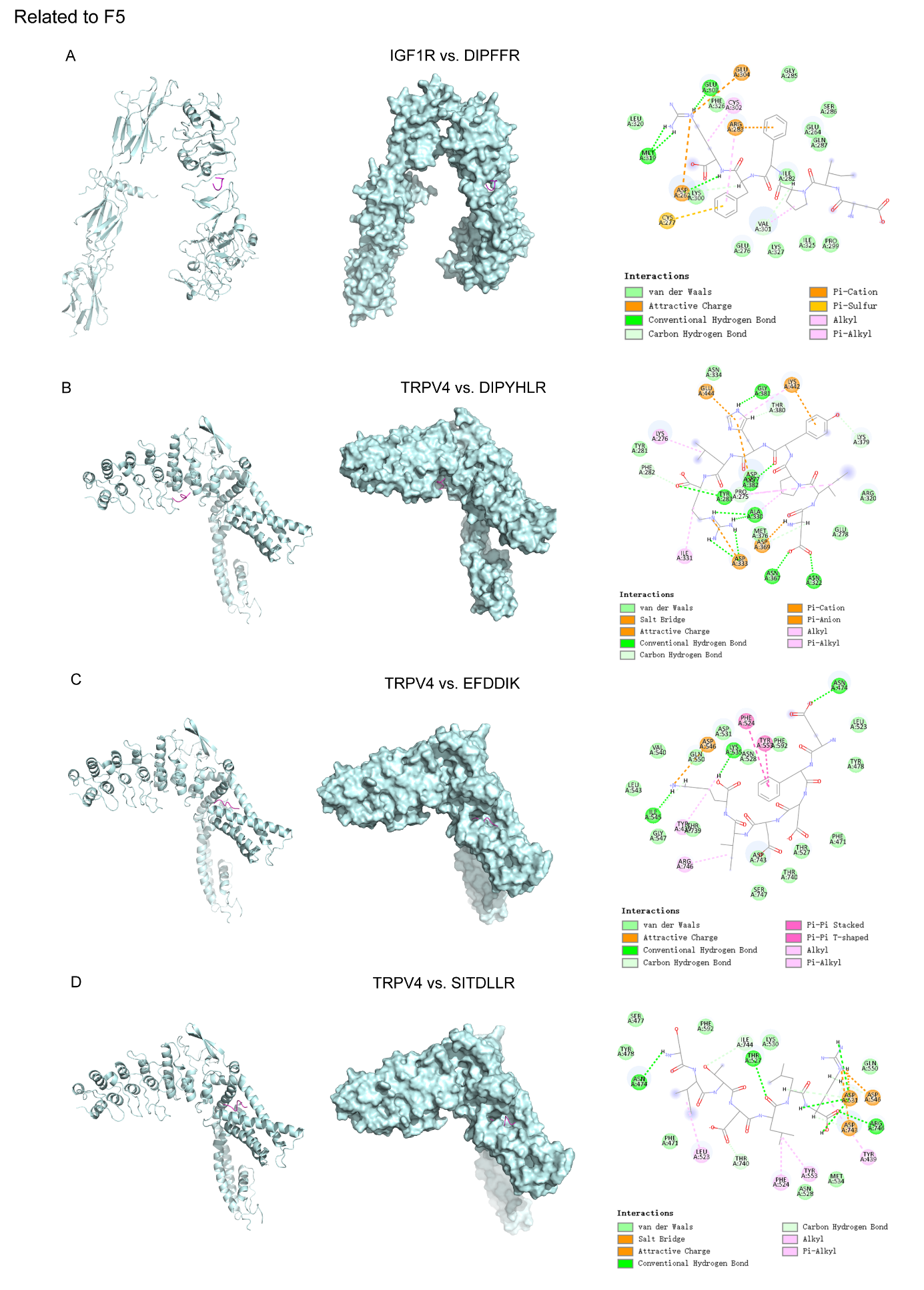


***Fig. S9.*** *Molecular interactions of (A) IGF1R between DIPFFR peptide, (B) TRPV4 between DIPYHLR peptide, (C) TRPV4 between EFDDIK peptide, (D) TRPV4 between SITDLLR.*


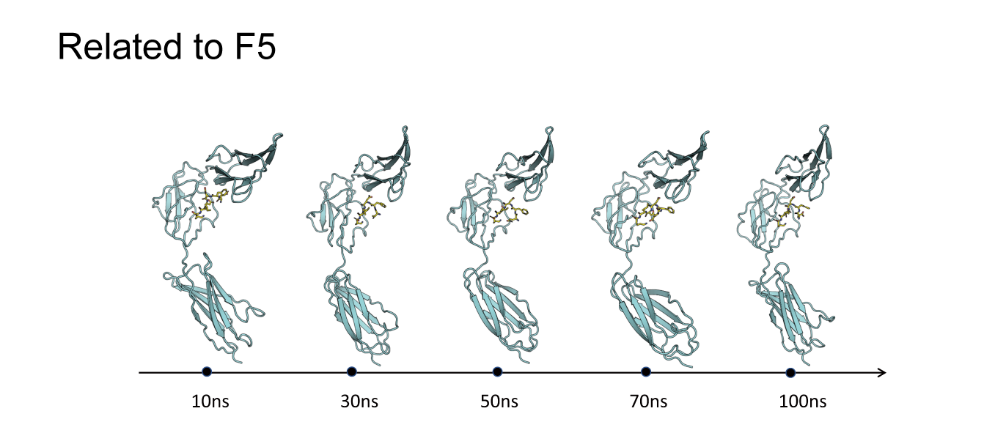


***Fig. S10.*** *Molecular dynamics simulation of time-dependent conformational changes of the complex at 10, 30, 50, 70, and 100 ns.*


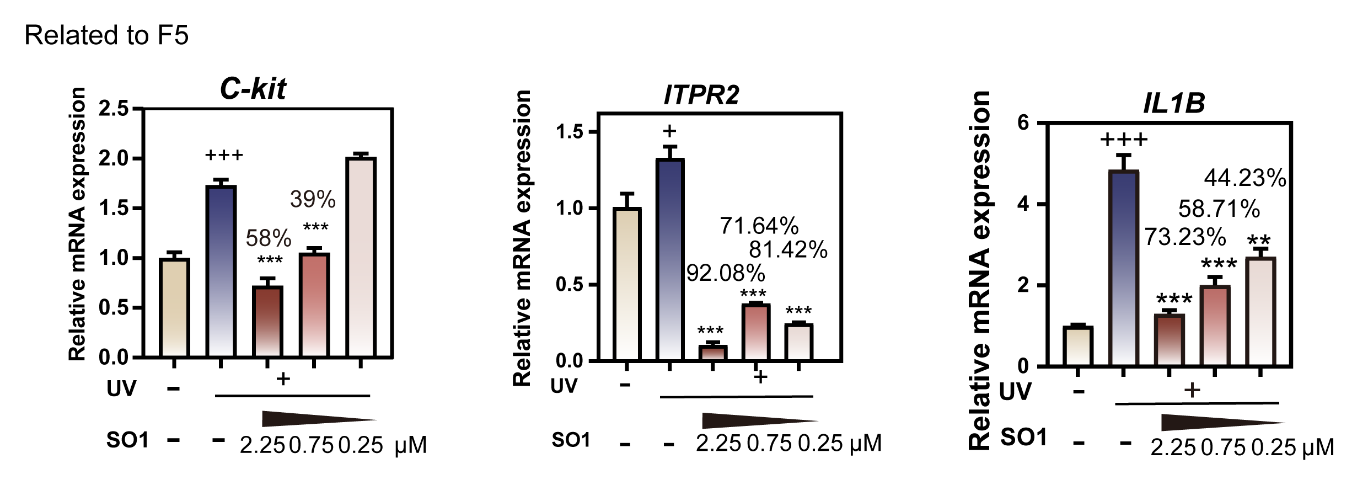


***Fig. S11.*** *qPCR analysis of C-kit, ITPR2 and IL1β.*


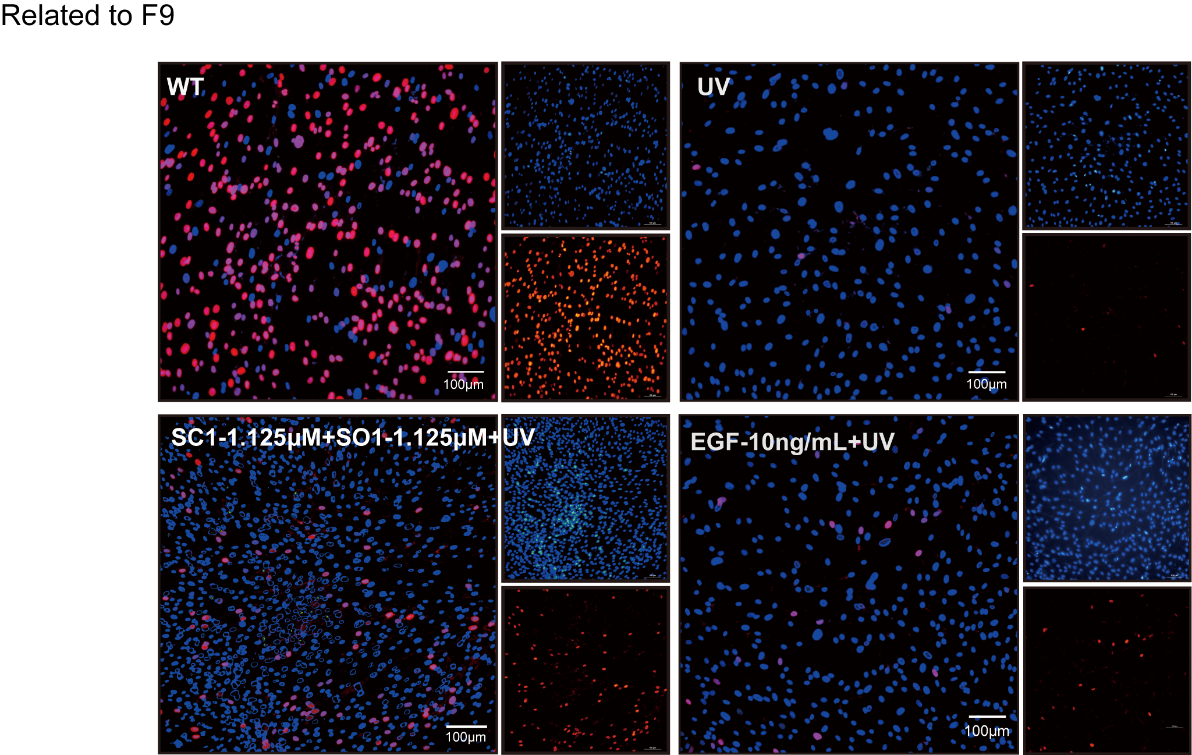


***Fig. S12.*** *Effects of different peptides on EdU staining in fibroblasts under UV irradiation. Scale bar = 100μm.*


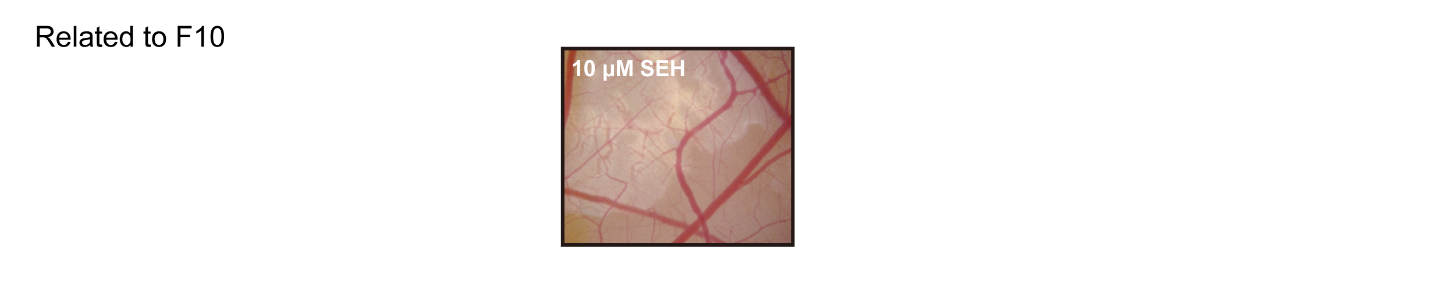


***Fig. S13.*** *Photographic images of CAM exposed to 10 μM SEH*.


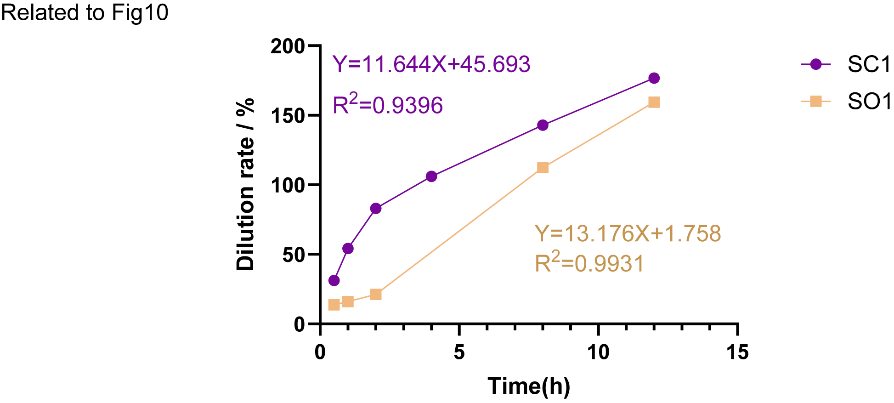


***Fig. S14.*** *Dilution rate of SO1 and SC1 peptides.*

REFERENCESUncategorized References

1. Kroemer, G., A.B. Maier, A.M. Cuervo, et al., "From geroscience to precision geromedicine: Understanding and managing aging." *Cell* (2025): 2043-2062. <http://doi.rog/10.1016/j.cell.2025.03.011>

2. Lopez-Otin, C., M.A. Blasco, L. Partridge, et al., "Hallmarks of aging: An expanding universe." *Cell* (2023): 243-278. <http://doi.rog/10.1016/j.cell.2022.11.001>

3. Yang, M., S. Wu, J. Zhang, et al., "Immunotherapies for Aging and Age-Related Diseases: Advances, Pitfalls, and Prospects." *Research (Wash D C)* (2025): 0866. <http://doi.rog/10.34133/research.0866>

4. Geng, L., J. Ping, R. Wu, et al., "Systematic profiling reveals betaine as an exercise mimetic for geroprotection." *Cell* (2025): 5426-5428. <http://doi.rog/10.1016/j.cell.2025.07.030>

5. Austin, E., A.N. Geisler, J. Nguyen, et al., "Visible light. Part I: Properties and cutaneous effects of visible light." *J Am Acad Dermatol* (2021): 1219-1231. <http://doi.rog/10.1016/j.jaad.2021.02.048>

6. Fisher, G.J., Z.Q. Wang, S.C. Datta, et al., "Pathophysiology of premature skin aging induced by ultraviolet light." *N Engl J Med* (1997): 1419-28. <http://doi.rog/10.1056/NEJM199711133372003>

7. Geisler, A.N., E. Austin, J. Nguyen, et al., "Visible light. Part II: Photoprotection against visible and ultraviolet light." *J Am Acad Dermatol* (2021): 1233-1244. <http://doi.rog/10.1016/j.jaad.2020.11.074>

8. Memon, F.U., C. Li, S. Ahmad, et al., "Efficiency of microbial fermentation on microbial shifts, enzymatic activity, and transcriptions in black soldier fly larvae during the sugarcane waste conversion." *Environ Pollut* (2025): 126588. <http://doi.rog/10.1016/j.envpol.2025.126588>

9. Memon, F.U., Y. Zhu, Y. Cui, et al., "Gut microbial communities and transcriptional profiles of black soldier fly (Hermitia illucens) larvae fed on fermented sericulture waste." *Waste Manag* (2025): 158-168. <http://doi.rog/10.1016/j.wasman.2025.01.011>

10. Wang, Z.H., F.R. Zeng, J.Y. Zhang, et al., "Highly Harsh-Environment-Stable and Sustainable Multifunctional Silk Textiles Enabled by Programmable Underwater Robust yet Stimulus-Reversible Cation-pi Adhesion." *Research (Wash D C)* (2025): 0910. <http://doi.rog/10.34133/research.0910>

11. Lu, H., K. Xia, M. Jian, et al., "Mechanically Reinforced Silkworm Silk Fiber by Hot Stretching." *Research (Wash D C)* (2022): 9854063. <http://doi.rog/10.34133/2022/9854063>

12. Wang, B., O. Hasturk, U. Kumarasinghe, et al., "Temporary Nanoencapsulation of Human Intestinal Organoids Using Silk Ionomers." *Adv Healthc Mater* (2025): e2403176. <http://doi.rog/10.1002/adhm.202403176>

13. Wang, W., Z. Sun, Y. Xiao, et al., "Silk acid-tyramine hydrogels with rapid gelation properties for 3D cell culture." *Acta Biomater* (2024): 138-148. <http://doi.rog/10.1016/j.actbio.2024.08.027>

14. Yang, K., J. Zhang, C. Zhang, et al., "Hierarchical design of silkworm silk for functional composites." *Chem Soc Rev* (2025): 4973-5020. <http://doi.rog/10.1039/d4cs00776j>

15. Xia, Q., S. Li, and Q. Feng, "Advances in silkworm studies accelerated by the genome sequencing of Bombyx mori." *Annu Rev Entomol* (2014): 513-36. <http://doi.rog/10.1146/annurev-ento-011613-161940>

16. Sun, Y., W. Shi, Q. Zhang, et al., "Multi-Omics Integration to Reveal the Mechanism of Sericin Inhibiting LPS-Induced Inflammation." *Int J Mol Sci* (2022): <http://doi.rog/10.3390/ijms24010259>

17. Wang, R., Y. Wang, J. Song, et al., "A Novel Approach for Screening Sericin-Derived Therapeutic Peptides Using Transcriptomics and Immunoprecipitation." *Int J Mol Sci* (2023): <http://doi.rog/10.3390/ijms24119425>

18. Wang, F., H. Lei, C. Tian, et al., "An Efficient Biosynthetic System for Developing Functional Silk Fibroin-Based Biomaterials." *Adv Mater* (2025): e2414878. <http://doi.rog/10.1002/adma.202414878>

19. Deng, H., F. Wang, Y. Zhou, et al., "Biosynthesis of a dual growth factors (GFs) functionalized silk sericin hydrogel to promote chronic wound healing in diabetic mice." *Bioact Mater* (2025): 511-528. <http://doi.rog/10.1016/j.bioactmat.2025.06.017>

20. Zhou, H., Y. Zhou, H. Deng, et al., "Genetically engineered sericin hydrogels for the delivery of human adiponectin in treating ulcerative colitis in mice." *Acta Biomater* (2025): 216-233. <http://doi.rog/10.1016/j.actbio.2025.07.030>

21. Rittie, L. and G.J. Fisher, "UV-light-induced signal cascades and skin aging." *Ageing Res Rev* (2002): 705-20. <http://doi.rog/10.1016/s1568-1637(02)00024-7>

22. Huang, R.P., J.X. Wu, Y. Fan, et al., "UV activates growth factor receptors via reactive oxygen intermediates." *J Cell Biol* (1996): 211-20. <http://doi.rog/10.1083/jcb.133.1.211>

23. Yu, E., S.W. Oh, S.H. Park, et al., "The Pigmentation of Blue Light Is Mediated by Both Melanogenesis Activation and Autophagy Inhibition through OPN3-TRPV1." *J Invest Dermatol* (2025): 908-918 e6. <http://doi.rog/10.1016/j.jid.2024.07.034>

24. Zhang, L., H. Zeng, L. Jiang, et al., "Heat promotes melanogenesis by increasing the paracrine effects in keratinocytes via the TRPV3/Ca(2+)/Hh signaling pathway." *iScience* (2023): 106749. <http://doi.rog/10.1016/j.isci.2023.106749>

25. Dong, Z., Q. Xia, and P. Zhao, "Antimicrobial components in the cocoon silk of silkworm, Bombyx mori." *Int J Biol Macromol* (2023): 68-78. <http://doi.rog/10.1016/j.ijbiomac.2022.10.103>

26. Sun, S., M. Hood, L. Scott, et al., "Differential expression analysis for RNAseq using Poisson mixed models." *Nucleic Acids Res* (2017): e106. <http://doi.rog/10.1093/nar/gkx204>

27. Wu, T., E. Hu, S. Xu, et al., "clusterProfiler 4.0: A universal enrichment tool for interpreting omics data." *Innovation (Camb)* (2021): 100141. <http://doi.rog/10.1016/j.xinn.2021.100141>

28. Michaelis, L., M.L. Menten, K.A. Johnson, et al., "The original Michaelis constant: translation of the 1913 Michaelis-Menten paper." *Biochemistry* (2011): 8264-9. <http://doi.rog/10.1021/bi201284u>

29. Salomon‐Ferrer, R., D.A. Case, and R.C. Walker, "An overview of the Amber biomolecular simulation package." *Wiley Interdisciplinary Reviews: Computational Molecular Science* (2013): 198-210. <http://doi.rog/10.1002/wcms.1121>

30. Mark, P. and L. Nilsson, "Structure and dynamics of the TIP3P, SPC, and SPC/E water models at 298 K." *The Journal of Physical Chemistry A* (2001): 9954-9960. <http://doi.rog/10.1021/jp003020w>

31. Sagui, C. and T.A. Darden, "Molecular dynamics simulations of biomolecules: long-range electrostatic effects." *Annual Review of Biophysics and Biomolecular Structure* (1999): 155-179. <http://doi.rog/10.1146/annurev.biophys.28.1.155>

32. Hou, T., J. Wang, Y. Li, et al., "Assessing the performance of the MM/PBSA and MM/GBSA methods. 1. The accuracy of binding free energy calculations based on molecular dynamics simulations." *Journal of Chemical Information and Modeling* (2011): 69-82. <http://doi.rog/10.1021/ci100275a>

33. Genheden, S. and U. Ryde, "The MM/PBSA and MM/GBSA methods to estimate ligand-binding affinities." *Expert opinion on drug discovery* (2015): 449-461. <http://doi.rog/10.1517/17460441.2015.1032936>

34. Rastelli, G., A.D. Rio, G. Degliesposti, et al., "Fast and accurate predictions of binding free energies using MM‐PBSA and MM‐GBSA." *Journal of Computational Chemistry* (2010): 797-810.

35. Wang, W., C. Tan, L. Li, et al., "The China National GeneBank Sequence Archive (CNSA) 2024 update." *Hortic Res* (2025): uhaf036. <http://doi.rog/10.1093/hr/uhaf036>
